# Genomic Insights into the Glutathione S-Transferase Gene Family of Two Rice Planthoppers, *Nilaparvata lugens* (Stål) and *Sogatella furcifera* (Horváth) (Hemiptera: Delphacidae)

**DOI:** 10.1371/journal.pone.0056604

**Published:** 2013-02-14

**Authors:** Wen-Wu Zhou, Qing-Mei Liang, Yi Xu, Geoff M. Gurr, Yan-Yuan Bao, Xue-Ping Zhou, Chuan-Xi Zhang, Jiaan Cheng, Zeng-Rong Zhu

**Affiliations:** 1 State Key Laboratory of Rice Biology, Key Laboratory of Agricultural Entomology, Ministry of Agriculture; and Institute of Insect Sciences, Zhejiang University, Hangzhou, People's Republic of China; 2 Institute of Biotechnology, Zhejiang University, Hangzhou, People's Republic of China; 3 EH Graham Centre for Agricultural Innovation, Charles Sturt University, Orange, New South Wales, Australia; Natural Resources Canada, Canada

## Abstract

**Background:**

Glutathione S-transferase (GST) genes control crucial traits for the metabolism of various toxins encountered by insects in host plants and the wider environment, including insecticides. The planthoppers *Nilaparvata lugens* and *Sogatella furcifera* are serious specialist pests of rice throughout eastern Asia. Their capacity to rapidly adapt to resistant rice varieties and to develop resistance to various insecticides has led to severe outbreaks over the last decade.

**Methodology/Principal Findings:**

Using the genome sequence of *N. lugens*, we identified for the first time the complete GST gene family of a delphacid insect whilst nine GST gene orthologs were identified from the closely related species *S. furcifera. Nilaparvata lugens* has 11 GST genes belonging to six cytosolic subclasses and a microsomal class, many fewer than seen in other insects with known genomes. Sigma is the largest GST subclass, and the intron–exon pattern deviates significantly from that of other species. Higher GST gene expression in the *N. lugens* adult migratory form reflects the higher risk of this life stage in encountering the toxins of non-host plants. After exposure to a sub-lethal dose of four insecticides, chlorpyrifos, imidacloprid, buprofezin or beta-cypermethrin, more GST genes were upregulated in *S. furcifera* than in *N. lugens*. RNA interference targeting two *N. lugens* GST genes, *NlGSTe1* and *NlGSTm2,* significantly increased the sensitivity of fourth instar nymphs to chlorpyrifos but not to beta-cypermethrin.

**Conclusions/Significance:**

This study provides the first elucidation of the nature of the GST gene family in a delphacid species, offering new insights into the evolution of metabolic enzyme genes in insects. Further, the use of RNA interference to identify the GST genes induced by insecticides illustrates likely mechanisms for the tolerance of these insects.

## Introduction

Insects have evolved diverse adaptations to toxicants present in their environment, most importantly, plant allelochemicals and pesticides. When avoidance, driven by vision, olfaction or other sensory systems fails, a variety of metabolic systems serve to handle toxins and minimize ill effects [1]. The multifunctional enzyme superfamily glutathione S-transferase (GST, EC 2.5.1.18) is one of the most important metabolic adaptive traits in insects. Most GST enzymes catalyse intracellular conjugation between glutathione (GSH) and a wide range of exogenous and endogenous compounds, making them more soluble and easier to excrete [2]–[4]. Some GST enzymes also exhibit non-catalytic functions such as non-substrate ligand binding and intracellular transportation of chemicals and stress signal processing [5]–[7]. More than 200 GST genes have now been validated in eight insect genomes and grouped into two classes (cytosolic and microsomal) and six major cytosolic subclasses (Delta, Epsilon, Omega, Sigma, Theta and Zeta) based on their location, function or sequence relatedness [2], [8].

The brown planthopper, *Nilaparvata lugens* (Stål) and the white-backed planthopper, *Sogatella furcifera* (Horváth) are widely distributed in Asia and the South Pacific regions. They are the two most economically important rice pests in Asian countries and ongoing outbreaks constitute a serious threat to the sustainability of rice production [9]. *Nilaparvata lugens* is a typical monophagous herbivore of rice (*Oryza sativa* L.) and its wild relatives. In contrast, *S. furcifera* is oligophagous with its host range extending across many genera of Poaceae. A third economically important rice planthopper, the small brown planthopper, *Laodelphax striatellus* (Fallén), has a similar host range to *S. furcifera.* Outbreaks of these species, especially *N. lugens* and *S. furcifera,* have been frequent in Asian rice crops over the last decade. Crops are seriously damaged by sap sucking and tissue damage from oviposition. Moreover, as vectors for at least four plant viruses coupled with the capacity for long-distance migration, these pests are responsible for widespread disease-related yield losses [10].

Rice planthoppers possess a high capacity to overcome stresses, such as those from host plant resistance and insecticides, in the new environments to which they migrate [11]. The use of rice varieties bred for planthopper resistance and extensive spraying of insecticides in Asia has not prevented planthopper outbreaks; indeed the ubiquity of their use is likely to have accelerated planthopper adaptation [12]. After feeding on the *N. lugens*-resistant rice variety B5, GST gene expression was significantly enhanced in *N. lugens* [13]. Similarly, enzyme activity was induced in *S. furcifera* by feeding on the *S. furcifera*-resistant rice varieties N22 and ASD7 [14]. Resistance monitoring of *N. lugens* in the laboratory indicated that metabolic resistance plays a key role in insecticide resistance, especially under low dose selection pressure [15]. The peroxidase activity and elevated expression of GST proteins from pyrethroid-resistant *N. lugens* strains suggested their involvement in insecticide resistance [16]. Increased GST activity was also observed in *S. furcifera* strains exposed to fipronil and synergists [17]. A comprehensive profiling of the GST gene superfamily at the genome and transcriptional levels would, therefore, be valuable in understanding the metabolic adaptation of rice planthoppers.

From the transcriptome databases of *N. lugens* and *S. furcifera*, together with the newly finished genome data set of *N. lugens* (Zhang *et al.*, *N. lugens* genome project at Zhejiang University, China, unpublished), we identified 11 and 9 GST genes for *N. lugens* and *S. furcifera*, respectively. We analyzed the expression response of these genes when exposed to insecticides. The roles of some *N. lugens* GST genes in insecticide resistance were also profiled with a newly-developed RNA interference (RNAi) technique. Further, the induced capacity of GST gene families was tested to explore their roles in insecticide tolerance. Moreover, using RNAi technology, the potential functions of some GSTs were, for the first time, predicted in a hemipteran insect. Our study provides the first genomic-level insights into the GST gene family of a monophagous insect and supports the wider understanding of the evolution of insect metabolic systems.

## Results

### Identification and classification of *N. lugens* and *S. furcifera* GST genes

**Figure 1:**
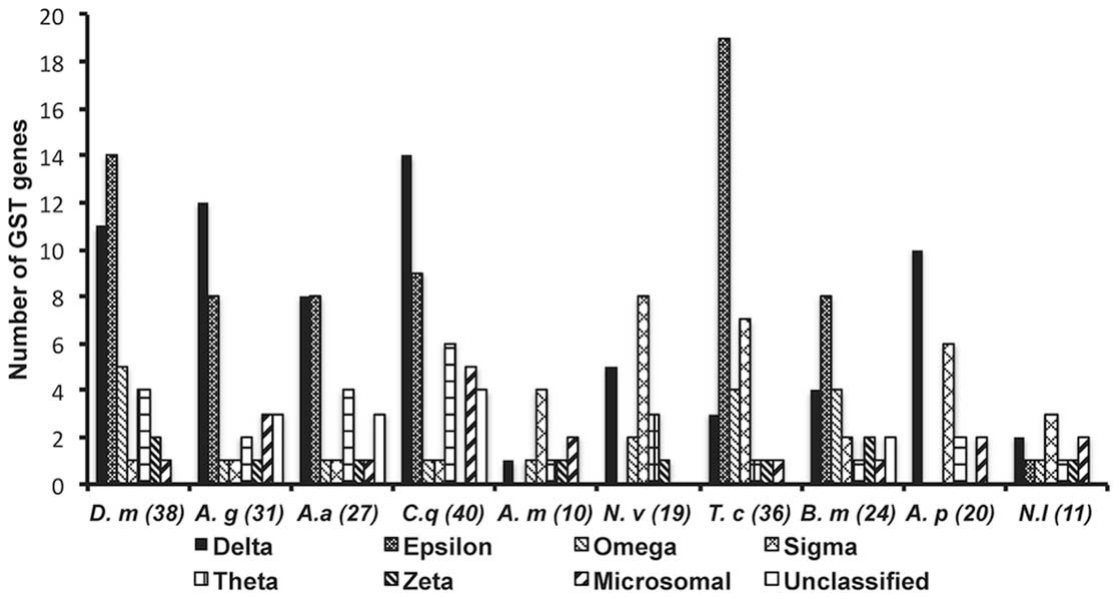
Number of validated glutathione S-transferase genes across insect genomes. *D. m*, *Drosophila melanogaster* [64]; *A. g*, *Anopheles gambiae* [65]; *A. a*, *Aedes aegypti* [66]; *C. q*, *Culex quinquefasciatus* [41]; *A. m*, *Apis mellifera* [67]; *N. v*, *Nasonia vitripennis* [68]; *T. c*, *Tribolium castaneum* [69]; *B. m, Bombyx mori* [70]; *A. p*, *Acyrthosiphon pisum* [71]; *N. l*, *Nilaparvata lugens*. Values in parentheses are species totals of all GST subclasses.

We found 13 GST-like sequences from the genome of *N. lugens*, and named them using the structure: *abbreviation for species+‘GST’+abbreviation for cystolic subclass+the order of its identification in the subclass*. Microsomal GST genes were designated *abbreviation for species+‘GSTm’+the order of its identification in the subclass*. Of the 13 GST-like sequences, 11 (*NlGSTd1*, *d2*, *e1*, *o1*, *s1*, *s2*, *s3*, *t1*, *z1*, *m1* and *m2*) appeared to be functional GST genes and two (*NlGSTo2* and *t2*) were probable pseudogenes (Table 1). *NlGSTs3* was incomplete with approximately 114 amino acids missing at the 3′ end compared with its orthologous gene *LsGSTs1*, due to an incomplete contig in the genome where *NlGSTs3* is located. In the transcriptome and EST databases of *N. lugens*, cDNAs segments were found for 11 GST genes but not for the two pseudogenes (Table 1). The Sigma subclass was the largest in *N. lugens* with three genes present (Table 1). Overall, however, *N. lugens* exhibited a reduction in GST gene numbers compared with other insects with complete genomes, especially for the Delta and Epsilon subclasses (Figure 1). A total of only three genes was identified in these two subclasses in *N. lugens* (*NlGSTd1*, *d2* and *e1*) (Figure 1). In contrast, the equivalent number in other insects from Diptera, Coleoptera, Lepidoptera and the hemipteran *Acyrthosiphon pisum*, is between 10 and 25. Whilst the hymenopteran *Nasonia vitripennis* shows a similar paucity of Delta and Epsilon subclass genes, it is relatively rich in Sigma subclass genes (Figure 1). In *S. furcifera*, nine genes (*SfGSTd1*, *d2*, *e1*, *o1*, *s1*, *t1*, *z1*, *m1* and *m2*) were identified from the three transcriptomes (Table 1). To confirm the accuracy of the gene identification, all genes were PCR-amplified and cloned, and their predicted sequences were deposited in GenBank with the accession numbers indicated in Table 1.

**Table 1:** GST genes of *Nilaparvata lugens* (Nl prefix) and *Sogatella furcifera* (Sf prefix).

GenBank	Gene	cDNA (bp)	Genome segments	GenBank	Gene	cDNA (bp)
JQ917469	*NlGSTd1*	790	scaffold105	JQ917481	*SfGSTd1*	772
JQ917467	*NlGSTd2*	832	scaffold740	JQ917483	*SfGSTd2*	759
JQ917470	*NlGSTe1*	812	scaffold1545	JQ917482	*SfGSTe1*	898
JQ917475	*NlGSTm1*	560	scaffold542	JQ917476	*SfGSTm1*	540
JQ917473	*NlGSTm2*	638	scaffold5696	JQ917478	*SfGSTm2*	567
JQ917471	*NlGSTo1*	911	scaffold863	JQ917479	*SfGSTo1*	779
JQ917468	*NlGSTs1*	824	scaffold1785+2705	JQ917480	*SfGSTs1*	795
JQ917474	*NlGSTs2*	704	scaffold76	JQ917477	*SfGSTt1*	844
JQ959596	*NlGSTs3*	330	scaffold4664	JQ917484	*SfGSTz1*	783
JQ917472	*NlGSTt1*	1120	scaffold3			
JQ946073	*NlGSTz1*	700	scaffold2415			

cDNA length given is for the cloned sequences.

The length of GST Open Reading Frames (ORF) was between 148 and 239 amino acids, with an identity ranging between 3.0% to 55.4% in *N. lugens* and 4.5% to 36.0% in *S. furcifera*. In *L. striatellus*, ORF length was 152 to 236 amino acids, and the identity 4.6% to 55.0% with a longer *LsGSTm* and shorter *LsGSTt* [18].

**Figure 2:**
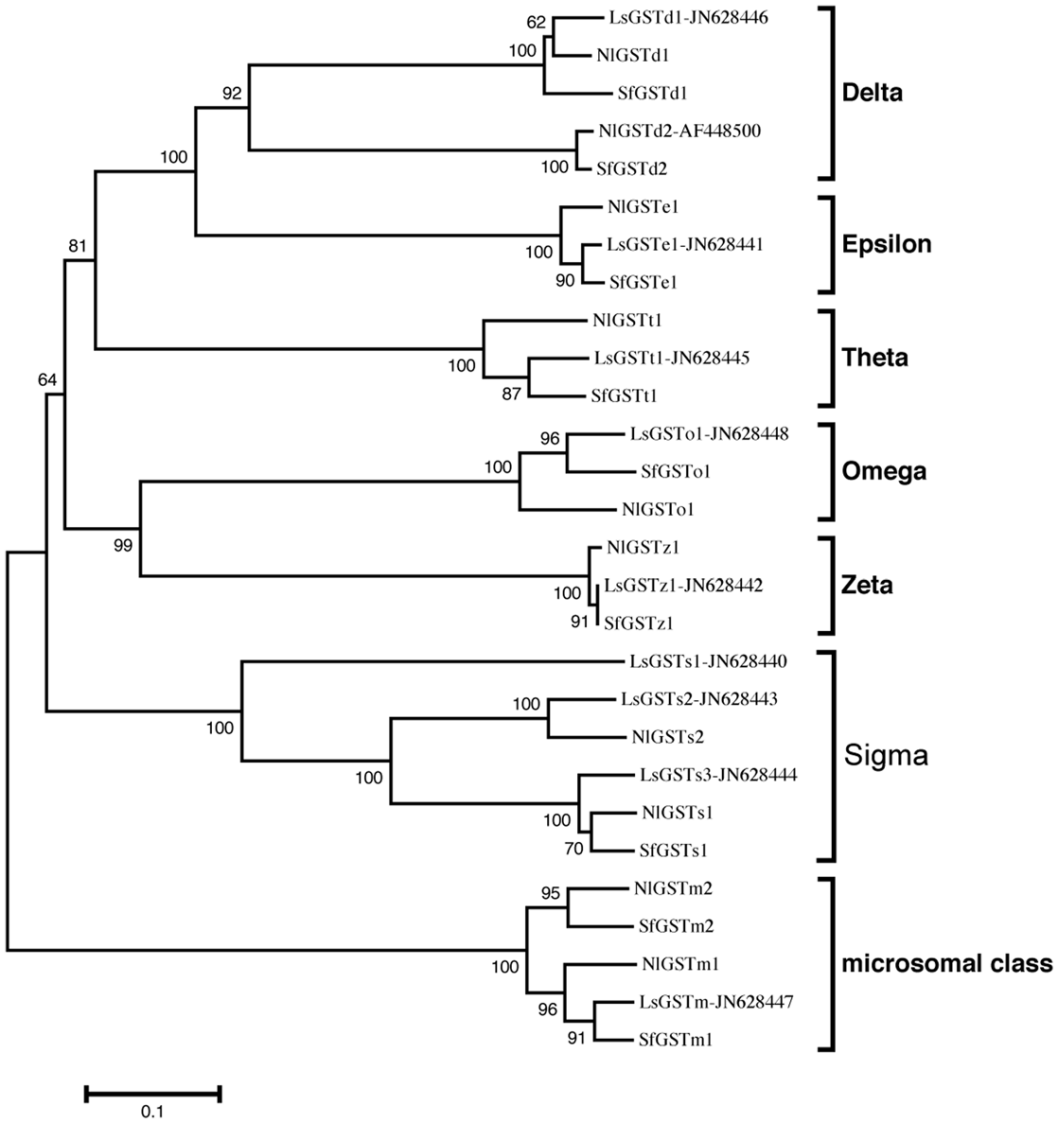
Neighbor-joining tree of glutathione S-transferase genes of the three rice planthoppers. *Nilaparvata lugens* (Nl prefix), *Sogatella furcifera* (Sf prefix) and *Laodelphax striatellus* (Ls prefix).

The three planthoppers, although each from different genera, display high similarities in GST gene sequences. All six subclasses and the microsomal class were consistently represented. To determine orthologous/paralogous relationships, a neighbor-joining tree based on the amino acid sequences was constructed (Figure 2). There were seven clearly identified sets of orthologs (d1, e1, t1, o1, s1, z1 and m1) among the three planthopper species. Other clades did not have a representative in all species (d2, s2 and m2), which was caused by incomplete transcriptome data for *S. furcifera* and *L. striatellus*. Notably, *LsGSTs1* was contained in a single branch in the phylogenetic tree without any closely related orthologous genes in the other two planthopper species, suggesting a unique function arising from gene duplication (Figure 2). Among all GSTs, the highest level of identity (99.5%) was found among members of the Zeta subclass genes (*LsGSTz1* and *SfGSTz1*), while the lowest level (81.7%) was between members of the Sigma subclass genes (*NlGSTs1* and *SfGSTs1*). The maximum likelihood method *ω* ratios of the normalized non-synonymous substitution rate (*d*_N_) to the normalized synonymous substitution rate (*d*_S_) was used for testing variation in selection pressures and for positive selection [19]. The *ω* ratios for all analyzed subclasses were smaller than 0.1 for nearly all gene groups compared, indicating purifying selection had occurred in all three species (Table 2). Among these orthologs, the Zeta subclass representatives (*NlGSTz1*, *LsGSTz1* and *SfGSTz1*) had the smallest mean *ω* ratio (Table 2), suggesting strong negative selection resulted from the initial importance of this GST gene subclass in three planthoppers. In contrast, the three Sigma subclass representatives (*LsGSTs3*, *NlGSTs1*, *SfGSTs1*) had the largest mean *ω* ratio (Table 2), indicating weaker negative selection.

**Table 2:** The dN/dS ratio and amino acid identity of homologous genes between three planthopper species, *L. striatellus*, *N. lugens* and *S. furcifera*.

	*LsGSTd1*	*NlGSTd1*	*SfGSTd1*		*LsGSTs3*	*NlGSTs1*	*SfGSTs1*
*LsGSTd1*				*LsGSTs3*			
*NlGSTd1*	*93.6*/0.042			*NlGSTs1*	*86.4*/0.093		
*SfGSTd1*	*92.7*/0.065	*91.8*/0.068		*SfGSTs1*	*88.9*/0.11	*81.7*/0.091	

	*LsGSTe1*	*NlGSTe1*	*SfGSTe1*		*LsGSTt1*	*NlGSTt1*	*SfGSTt1*
*LsGSTe1*				*LsGSTt1*			
*NlGSTe1*	*93.1*/0.034			*NlGSTt1*	*93.6*/0.062		
*SfGSTe1*	*96.3*/0.023	*92.7*/0.024		*SfGSTt1*	*93.1*/0.091	*92.6*/0.047	

	*LsGSTo1*	*NlGSTo1*	*SfGSTo1*		*LsGSTz1*	*NlGSTz1*	*SfGSTz1*
*LsGSTo1*				*LsGSTz1*			
*NlGSTo1*	*87.3*/0.05			*NlGSTz1*	*98.6*/0.0124		
*SfGSTo1*	*89.2*/0.073	*83.7*/0.066		*SfGSTz1*	*99.5*/0.0423	*99.1*/0.0077	

Italics indicate the amino acid identities of homologous genes between different species.

**Figure 3:**
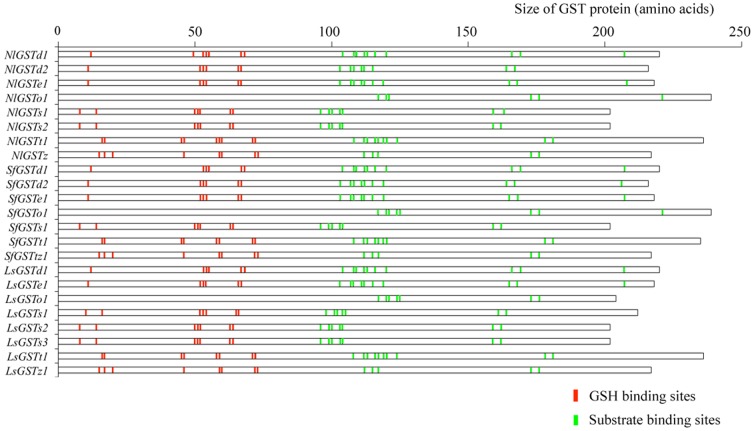
GSH and substrate binding sites of glutathione S-transferase genes across three planthopper species. *Nilaparvata lugens* (Nl prefix), *Sogatella furcifera* (Sf prefix), *Laodelphax striatellus* (Ls prefix). The short vertical lines represent functionally conserved residues of GST genes among insect species.

To elucidate the structure and putative biochemical role of the GSTs in the three planthoppers, GSH and substrate binding sites were analyzed using the NCBI CD-search program (Figure 3 and Table S2). In insects, the NH_2_-terminus of GST enzymes is the most highly conserved region because it contains residues important in the binding and activation of GSH, whereas the COOH-terminus contains the majority of the residues conferring substrate specificity [20]. In our study, the GSH binding sites of *N. lugens* were more strongly conserved among orthologous genes compared with substrate binding sites. For instance, Delta subclass genes (*NlGSTd1*, *SfGSTd1* and *LsGSTd1*) shared the same GSH binding sites: 12S, 53H, 54T, 55I, 67D, 68S; Epsilon subclass genes (*NlGSTe1*, *SfGSTe1* and *LsGSTe1*) shared 11S, 52H, 53T, 54I, 66D, 67S; Sigma subclass genes (*NlGSTs1*, *SfGSTs1* and *LsGSTs3*) shared 8Y, 14L, 50Q, 51V, 52P, 63Q, 64S; and Zeta subclass genes (*NlGSTz1*, *SfGSTz1* and *LsGSTz3*) shared 15S, 17C, 20R, 46Q, 59Q, 60V, 72E, 73S. The conservation of GSH binding sites across the three planthopper species indicates their important enzyme function, whilst variations reflect evolutionary divergence. For instance, only *NlGSTt1* had an additional site with proline (60) compared with its orthologs.

### Comparison of GST genes from *N. lugens*, *S. furcifera* and other insects

**Figure 4:**
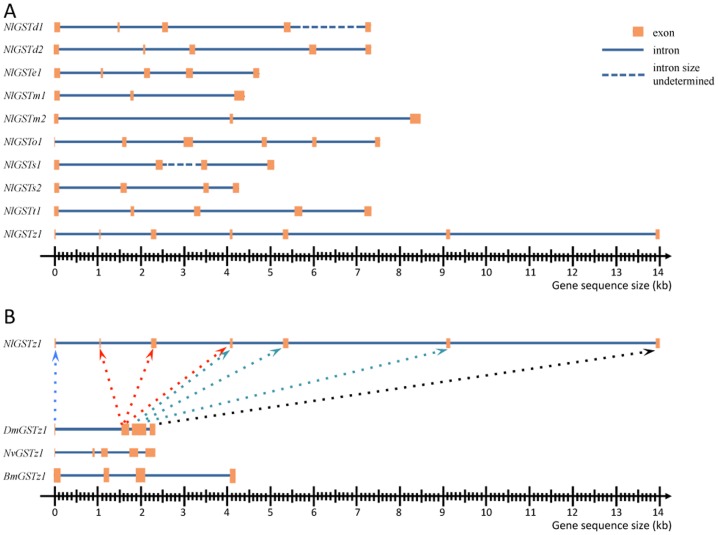
Structure of GST genes in insects was drawn using the genomic coordinates of each GST gene ORF region on its scaffold. A, Structure of GST genes in *N. lugens*; B, Comparison of Zeta subclass GST genes from four insects. (*Nilaparvata lugens* (Nl prefix), *Drosophila melanogaster* (Dm prefix), *Nasonia vitripennis* (Nv prefix), *Bombyx mori* (Bm prefix). Horizontal lines represent the scaffold regions which contain the GST gene. Arrow-head broken lines show the evolvement of exon segments between *N. lugens* and *D. melanogaster.*

In *N. lugens*, many introns were found not only in the 5′-most part of the ORF, but also distributed across the whole gene. The GST genes of *N. lugens* were distributed across 11 scaffolds of genome sequences ranging from 4355 bp (*NlGSTm1*) to 13996 bp (*NlGSTz*) (Figure 4A), and the average length of the GST genes (introns+exons) was 6889.9 bp. The putative intron/exon of *NlGSTs3* could not be analyzed completely due to missing 3′ end sequences, and only 2 exons were found in the 5′ end segment. With 2 to 6 introns, the introns of *N. lugens* GST genes were longer and more numerous than in their counterparts *D. melanogaster*, *A. gambiae* and *C. quinquefasciatus* (Figure 4B). GST gene intron lengths ranged from 614 bp to 4710 bp with an average length of 1746.14 bp (n = 36 introns) for *N. lugens* (Table S3).

**Figure 5:**
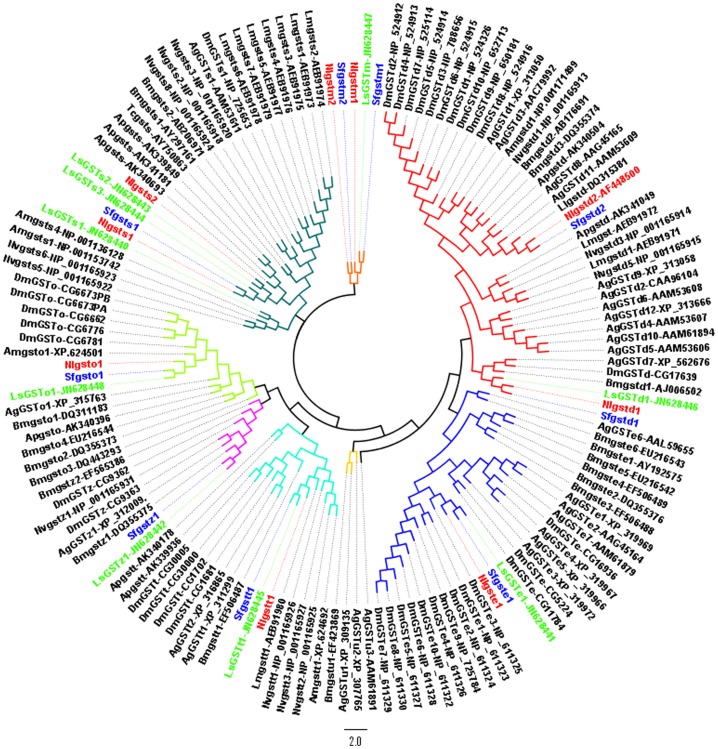
Phylogenetic relationships of 145 GST proteins from 12 insect species. *Anopheles gambiae* (Ag, 27), *Apis mellifera* (Am, 5), *Bombyx mori* (Bm, 19), *Drosophila melanogaster* (Dm, 36), *Sogatella furcifera* (Sf, 9), *Nasonia vitripennis* (Nv, 11), *Locusta migratoria manilensis* (Lm, 10), *Acyrthosiphon pisum* (Ap, 8), *Toxoptera citricida* (Tc, 1), *Lygus lineolaris* (Ll, 1), *Nilaparvata lugens* (Nl, 9) and *Laodelphax striatellus* (Ls, 9). Branches of the genes from the same subclass are indicated by the same color.

Bootstrap scores supported the higher level structure of the phylogeny analysis of GST genes in planthoppers and nine other insect species (Table S4) and allowed all subclasses to be distinguished. There was greater similarity among hemipteran Sigma subclass GST genes (*N. lugens*, *S. furcifera*, *L. striatellus*, *A. pisum* and *T. citricida*) compared with the Sigma GSTs of other insect orders (Figure 5). In contrast, genes encoding the Theta, Delta and Epsilon GSTs were more diverse, suggesting varied functional roles in the metabolism of xenobiotics.

**Figure 6:**
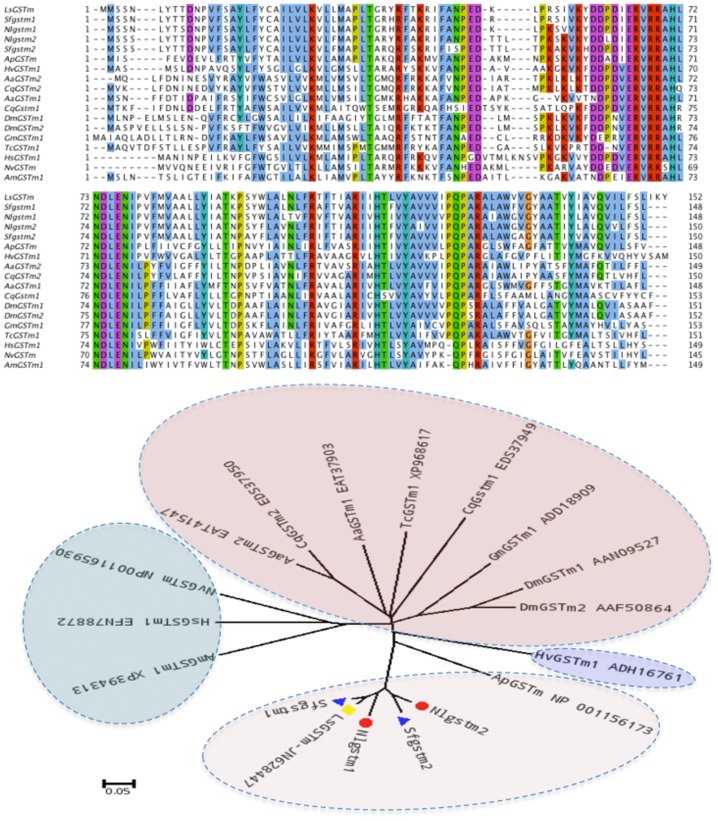
Phylogenetic relationships and amino acid comparison of microsomal GST proteins from 13 insect species. *Acyrthosiphon pisum* (Ap), *Culex quinquefasciatus* (Cq), *Aedes aegypti* (Aa), *Heliothis virescens* (Hv), *Harpegnathos saltator* (Hs), *Nasonia vitripennis* (Nv), *Apis mellifera* (Am), *Tribolium castaneum* (Tc), *Drosophila melanogaster* (Dm), *Sogatella furcifera* (Sf), *Nasonia vitripennis* (Nv), *Nilaparvata lugens* (Nl) and *Laodelphax striatellus* (Ls).

A neighbor-joining phylogenetic analysis of microsomal GSTs proteins from 13 insect species revealed that they cluster into 4 groups (Figure 6). Most groups were comprised of species from the same order except one that included Diptera and Coleoptera species. A highly conserved region with the amino acids ERVRRAHLNDLENI was present in all insect MGGSTs analyzed (Figure 6), indicating its potential importance as a functional domain.

### Transcription profiling of GST genes in different life-stages and tissues

**Figure 7:**
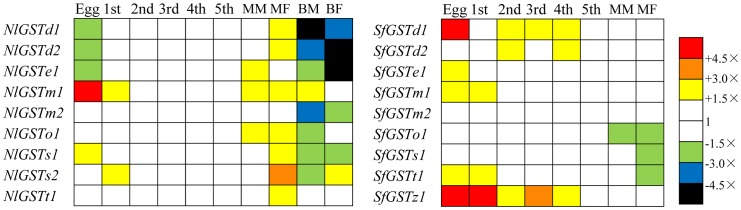
Constitutive transcription profiles of nine *N. lugens* GSTs (left) and nine *S. furcifera* GSTs (right) across different life stages, sexes and wing-forms (right). Egg (E), 1st instar-nymph (1st), 2nd instar-nymph (2nd), 3rd instar-nymph (3rd), 4th instar-nymph (4th), 5th instar-nymph (5th), macropterous adult male (MM), macropterous adult female (MF), brachypterous adult male (BM), brachypterous adult female (BF). Transcription levels are shown as mean fold transcription relative to 5th instar-nymph. Yellow, orange and red indicate significant over-transcription, whilst green, blue and black indicate significant under-transcription (Mann-Whitney test *P*-value<0.05. Data were converted by SPSS v19.0). White indicates no significant transcription variation.

In insects, the expression of GST genes is life-, stage-, sex- and tissue-specific and influenced by internal and external factors including physiological condition, diet and exposure to toxins [21]–[23]. In *N. lugens*, GST gene expression levels were generally highest in macropterous adults, but lower in eggs and lower still in nymphs. GST mRNA levels were the lowest overall in brachypterous adults. In *S. furcifera*, GST mRNA expression levels were highest in eggs, followed by nymphs. Macropterous adults displayed similar GST expression levels as in nymphs, except for *SfGSTo1*, *SfGSTs1* and *SfGSTt1* which were slightly downregulated (Figure 7).

**Figure 8:**
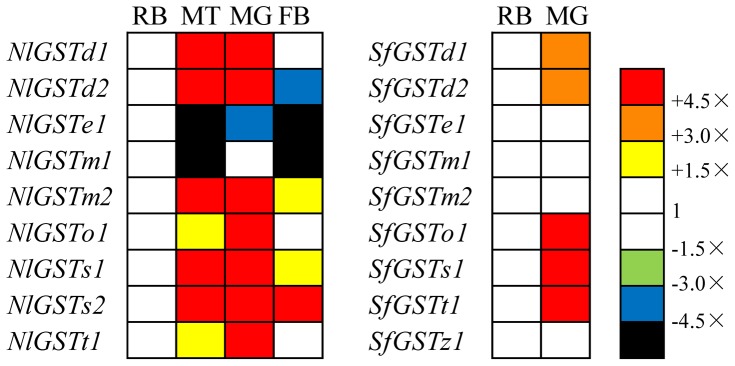
Constitutive transcription profiles of nine *N. lugens* GSTs and nine *S. furcifera* GSTs across different nymph tissues. Tissues analyzed were: remaining body (ie without midgut, fatbody and Malpighian tubule) (RB), fatbody (FB), midgut (MG), Malpighian tubules (MT). Transcription levels are expressed as mean fold transcription relative to remaining body. Yellow, orange and red indicate significant over-transcription, whilst green, blue and black indicate significant under-transcription (Mann-Whitney test *P*-value<0.05. Data were converted by SPSS v19.0). White indicates no significant transcription variation.

The midgut and Malpighian tubules are important insect metabolic centers [24]. In *N. lugens*, seven GST genes (*NlGSTd1, d2, m2, o1, s1, s2, t1*) were highly expressed in these two tissues (Figure 8). In *S. furcifera*, five GST genes (*SfGSTd1*, *d2*, *o1*, *s1*, *t1*) were also highly expressed in the midgut. The Epsilon subclass genes *NlGSTe1* showed higher mRNA level in the remaining bodies than in the metabolic centers, while *SfGSTe1* showed similar mRNA level between the midguts and the remaining bodies (Figure 8). Moreover, two homologous genes, *NlGSTm1* and *SfGSTm1* (with a high amino acid identity of 90.5), also had a different tissue expression pattern. In *N. lugens*, two genes (*NlGSTm2* and *s1*) were more highly expressed in three tissues midgut, Malpighian tubules and fat body, and *NlGSTs2* was highly expressed (more than 4.5-fold) across all the three tissues. In contrast, the mRNA level for gene *NlGSTd2* was much lower in the fatbody, though it was highly expressed in the midgut and Malpighian tubules.

### Transcription profiling in nymphs exposed to insecticides

**Figure 9:**
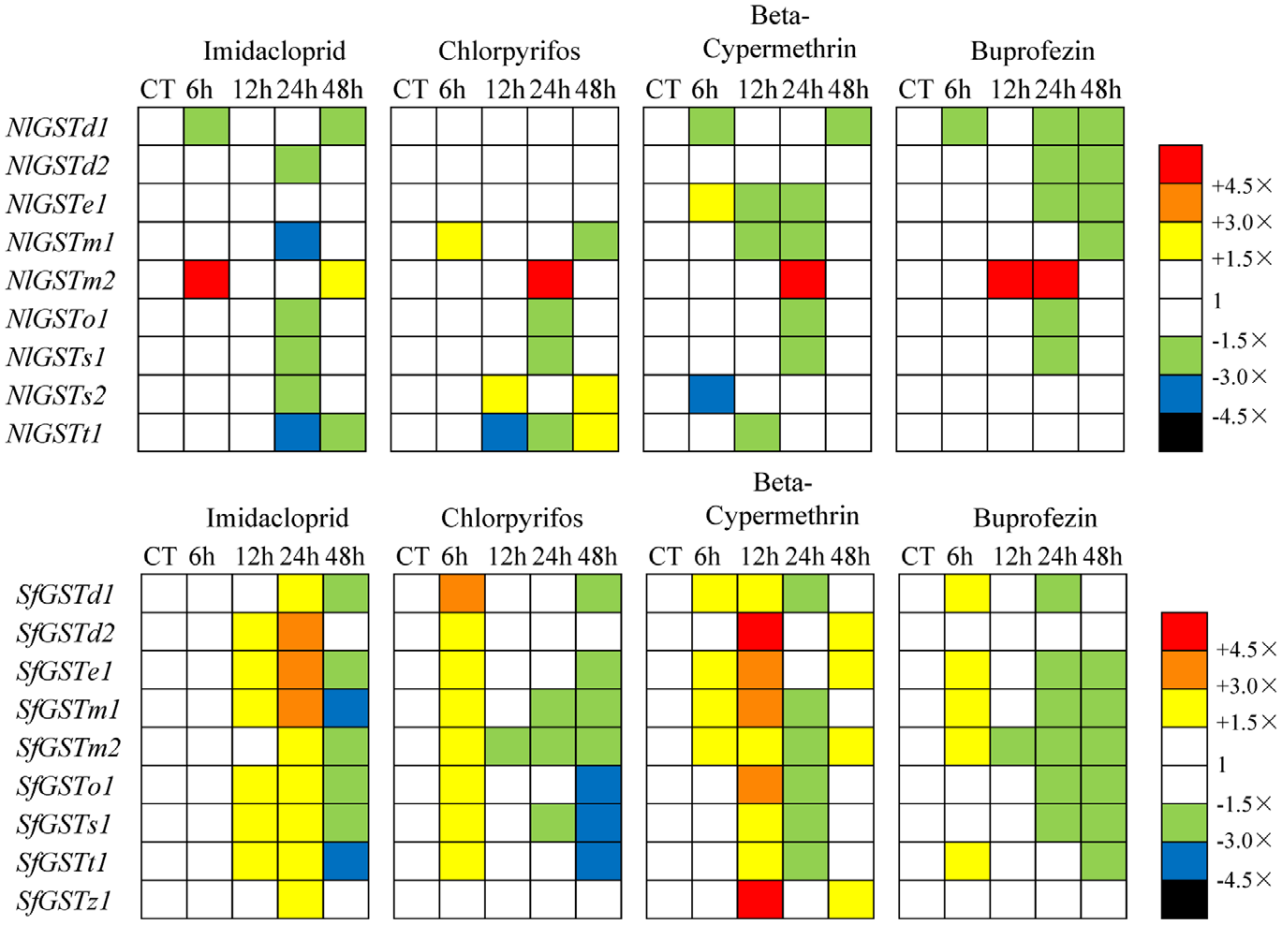
Transcription profiles of nine *N. lugens* GSTs and nine *S. furcifera* GSTs in third instar-nymphs exposed to sub-lethal concentrations of four different insecticides, Imidacloprid, chlorpyrifos, beta-cypermethrin and buprofezin. For each time point (6, 12, 24 and 48 hours), transcription levels are expressed as mean fold transcription relative to controls (unexposed nymphs) (CT). Yellow, orange and red indicate significant over-transcription, whilst green, blue and black indicate significant under-transcription (Mann-Whitney test *P*-value<0.05. Data was converted by SPSS v19.0). White indicates no significant transcription variation.

The expression pattern of GST mRNAs following insecticide treatment differed markedly between the two planthoppers studied. In *S. furcifera*, almost all the GST mRNAs were induced at at least one time point after insecticide application (Figure 9). In general, insecticides provoked the induction of a larger set of GST genes in *S. furcifera*, while in *N. lugens* the induction of GST genes was more restricted to time and insecticide type (Figure 9). Of the five over-expressed *N. lugens* GST genes, *NlGSTm2* was the only one over-expressed by all insecticides. The remaining GST mRNAs were each induced by a single insecticide, but at much lower level.

The Delta and Epsilon GST subclasses are the two largest GST subclasses implicated in the metabolism of xenobiotics [7], [25], [26]. High peroxidase activity level of the Delta subclass gene *NlGSTd2* (AF448500) was reported in a pyrethroid-resistant *N. lugens* strains [16], [27]. Two Delta subclass genes of *N. lugens, NlGSTd1* and *NlGSTd2,* did not show induced over-expression to all the insecticides tested. In *S. furcifera* however, the two Delta GSTs, *SfGSTd1* and *d2* showed significant induction (more than 4.5-fold) after treatment with the synthetic pyrethroid beta-cypermethrin (Figure 9).

The Sigma subclass has a wide taxonomic distribution and may be essential for housekeeping-related roles in insects such as the antioxidant function of the GST-2 enzyme in *D. melanogaster* [28]. In *N. lugens*, *NlGSTs2* was activated at 12 to 48 hours by chlorpyrifos, while in *S. furcifera, SfGSTs1* was activated by imidacloprid (12 to 24 hours), chlorpyrifos (6 hours) and beta-cypermethrin (6 hours). GSTs from the Theta subclass have a peroxidase function and in *Anopheles cracens*, *AcGSTT1-1* has been found to bind to organophosphates [29]. In *N. lugens*, *NlGSTt1* was also insensitive to most insecticides except for chlorpyrifos at 48 hours; while *SfGSTt1* was sensitive to all the insecticides tested in *S. furcifera*. Enzymes encoded by Zeta subclass genes are abundant at the protein levels in the permethrin-resistant strain of *B. mori*, suggesting their role in detoxification of chlorinated xenobiotics [30]. In *S. furcifera*, *SfGSTz1* was insensitive to the two insecticides chlorpyrifos and buprofezin, while it was sensitive to imidacloprid (24 hours) and beta-cypermethrin (12 and 48 hours).

### RNA interference of *N. lugens* GST gene expression by double-stranded RNA injection and its effect on insecticide tolerance

**Figure 10:**
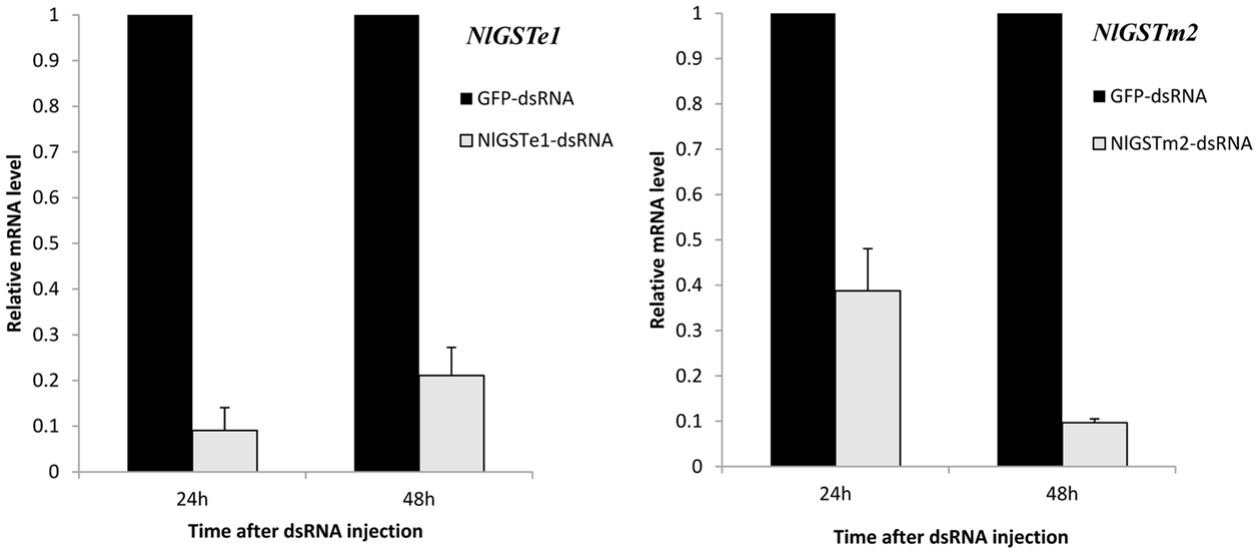
mRNA level of GSTs after RNA interference. GFP-dsRNA, nymph injected with dsRNA of Green fluorescent protein (GFP) gene; *NlGSTe1*-dsRNA, nymph injected with dsRNA of *NlGSTe1*; *NlGSTm2*-dsRNA, nymph injected with dsRNA of *NlGSTm2.*

**Figure 11:**
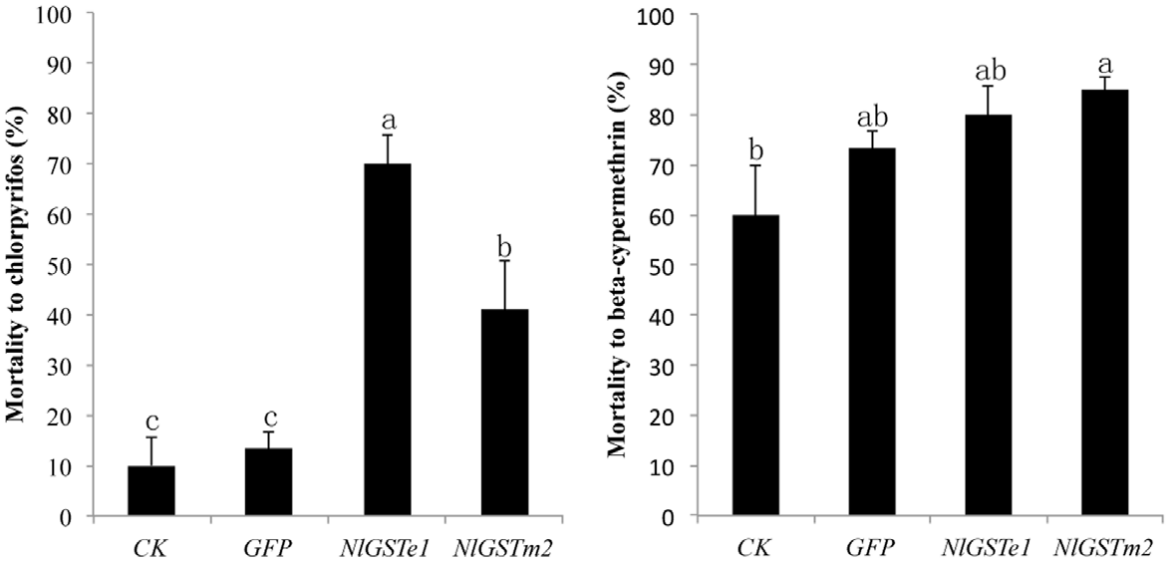
Mortality of *N. lugens* after RNA interference of GST genes to two insecticides. CK, nymph without any injection; GFP, nymph injected with dsRNA of Green fluorescent protein (GFP) gene; *NlGSTe1*, nymph injected with dsRNA of *NlGSTe1*; *NlGSTm2*, nymph injected with dsRNA of *NlGSTm2*. Values with different letters are significantly different as determined using a one-way ANOVA (Duncan's multiple range test, *P*<0.05).

In *N. lugens*, RNAi has been proven effective in gene silencing [31]–[33]. The recent discovery of the presence of RNAi pathway-related genes (*Nlsid-1* and *Nlaub*) in *N. lugens* also explained the success of double-stranded RNA (dsRNA)-mediated RNAi for different genes in former studies [34]. To test the function of GSTs in *N. lugens*, we injected dsRNAs targeting the *NlGSTe1* and *NlGSTm2* genes. These two genes were selected because their expression was induced either by exposure to a single insecticide (*NlGSTe1*, permethrin) or by all four insecticides tested (*NlGSTm2*). Moreover, *NlGSTe1* belongs to the Epsilon subclass which is involved in the metabolism of environmental chemicals [25], [26]. *NlGSTm2* was selected as more knowledge regarding the function of insect microsomal GSTs is needed. After injection both genes displayed a significant decrease in expression at 24 hours, with effects still evident at 48 hours (Figure 10). The decrease in *NlGSTe1* mRNA reached 90% at 24 hours, and 80% at 48 hours, compared with GFP dsRNA-injected controls. The decrease in *NlGSTm2* transcripts reached 60% at 24 hours, and 90% at 48 hours compared with controls. GST enzyme activity also exhibited a significant decrease at 36 hours after treatment with *NlGSTe1* dsRNA but not with *NlGSTm2* dsRNA (Figure S1), which may reflect the different substrate binding properties of these two enzymes. The knockdown of *NlGSTe1* or *NlGSTm2* does not have a direct effect on the viability of *N. lugens* 3rd instar nymphs, since no significant differences were observed in the mortality of dsRNA-injected individuals (Figure S2). Sensitivity to chlorpyrifos was significantly increased at 24 hours after injection of GST dsRNAs, as the mortality of fourth instar nymphs increased by 60% when *NlGSTe1* was silenced and by 30% when *NlGSTm2* was silenced (Figure 11).

## Discussion

Reflecting the metabolic roles of GST enzymes in insects, diet is likely to be a reason for the low number of GST genes observed in *N. lugens* compared with other insects for which a genome is available. *Nilaparvata lugens* is monophagous and exhibits impaired development or mortality within a few days on plant species other than rice [35]. Breeding programs in rice have focused chiefly on the improvement of yield [36] and this may have weakened antibiosis-based resistance in modern rice varieties, lessening the selection pressure on detoxification genes in insect pests such as *N. lugens*. Furthermore, compared with insects with mandibulate mouthparts (eg. Lepidoptera larvae), the suctorial mode of feeding by *N. lugens* restricts the diet to phloem contents, consisting primarily of water, sucrose (the only sugar) [37], free amino acids and inorganic constituents (Cl, K, Na, S and P) [38]. This diet contains fewer plant defence compounds and a restricted range of toxin types compared with other plant tissues such as foliage [39], [40]. It is interesting to note that *N. lugens* has few genes encoding GSTs of the Delta and Epsilon subclasses (2 and 1 genes respectively), enzymes which are recognized for their involvement in tolerance to plant defence compounds [41]. A relatively fewer cytochrome P450 enzymes in *N. lugens* compared with other insect species (Yan-yuan Bao, pers com.), also affects the function of crucial metabolic systems. The honeybee, *A. mellifera,* whose diet of honey and pollen consists primarily of water, soluble sugars, proteins and lipids, also has a low number of GST genes and is less heavily protected by toxins [42]. In contrast, a recent study of laboratory strains of the mosquito *Anopheles arabiensis,* with a broader diet, revealed a more flexible detoxification system with at least 8 GST genes (3 from Delta subclass) over-transcribed under the stress of toxin exposure [43].

The great number of orthologous GST gene groups in the three planthopper species suggests the radiation event or independent expansion of the GST gene family in these species may have occurred relatively recently. The similar diets (consisting mainly of rice as well as some other Poaceae species) and habitats (primarily rice-dominated) of the three planthoppers provide support for this theory. Planthoppers are likely to be exposed to a relatively narrow range of plant defence compounds. Orthologous genes of the Zeta subclass in the three planthopper species (*NlGSTz1*, *SfGSTz1* and *LsGSTz1*) had an average amino acid similarity of 99.07%. This is a higher level of similarity than reported for the two midges *Chironomus riparius* (87.5%) [44] and *C. tentans* (95.7%) [45].

The substrate binding sites of *N. lugens* GST genes appeared more diverse than the GSH binding sites. *LsGSTd1* had an alanine at position 113, compared with a glutamic acid in the *d1* orthologs of *N. lugens* and *S. furcifera*; *NlGSTe1* had a methionine at position 103 instead of glycine; *SfGSTt1* lacked a leucine at position 124; and *SfGSTz1* had a valine at position 173 instead of asparagine. Such diversity may reflect the selection pressures associated with differences in host plant range for the three planthoppers. We did not find any GSH binding domain or sites in the three Omega subclass genes (*NlGSTo1*, *SfGSTo1* and *LsGSTo1*), which may reflect a role in binding rather than in metabolizing of the xenobiotics, as shown in *A. cracens* [29].

Insect GST genes were previously hypothesized to be intronless which would allow rapid expression under stress [46]. With the accumulation of genome data, however, many insect GST genes were found to have 5′ introns [47]. The range of intron lengths for *D.* melanogaster, however, was much narrower and many Delta subclass genes in this species were intronless. In *A. gambiae*, the intron length ranged from 64 bp in Epsilon and Delta subclasses to 13937 bp in the Zeta subclass, with the majority of introns ranging from 50 to 100 bp [48]. Similar intron lengths were observed in *C. quinquefasciatus*, with 70% of introns being less than 100 bp and longer introns appeared to be enriched in the heterochromatin region of the chromosomes [41]. Low levels of homology between introns in the genomes of insect species indicate that these introns may have arisen quite recently [49]. In the current study for example, the length of introns in *N. lugens* GST genes differed markedly, despite significant conservation of the intron number and position among GST genes from the Sigma subclasses and microsomal class.

The life-stage expression patterns of GST genes among the planthoppers studied reflects the functional diversity of these genes within different species. In the current study, expression of GST genes in *N. lugens* was higher in macropterous adults than brachypterous adults, a pattern similar to that observed in a previous study of *L. striatellus* GST genes [18]. Migration by macropterous adults is an important biological phenomenon in planthoppers and long-distance migration, assisted by monsoon winds, results in landings in a wide range of vegetation types [50]. The increase in the GST enzymes in macropterous adults, especially in females that are essential in the establishment of new populations, would improve their capacity for survival by allowing feeding on a wide a range of rice genotypes and wild relatives. Additionally, an increase in GST enzymes would enable macropterous adult planthoppers to survive intoxication from attempted feeding bouts on non-host plants. In contrast, brachypterous adults develop within populations that are established on suitable host plants and so are less likely to encounter non-host plant toxins.

In *N. lugens*, seven GST genes (*NlGSTd1*, *d2*, *e1*, *m2*, *o1*, *s1* and *t1*) showed similar mRNA levels across the five nymph stages, compared with only four in *S. furcifera* (*SfGSTe1*, *m2*, *o1* and *s1*) (Figure 7) and three in *L. striatellus* (*LsGSTd1*, *m* and *o1*) [18]. Moreover, compared with *SfGSTd1*, *NlGSTd1* showed a similar expression pattern across the six life stages (five nymph stages and the macropterous adult male stages) (Figure 7) and is similar to the *LsGSTd1* in *L. striatellus* [18]. This difference in GST expression could be due to the breadth of host plants used by the three species, with *N. lugens* being monophagous, while *L. striatellus* and *S. furcifera* are polyphagous [51].

The GST mRNAs of the Delta and Epsilon subclasses in *N. lugens* were expressed at higher levels in nymphs than in brachypterous adults, a pattern previously reported in *L. striatellus* [18]. Differences in the GST mRNAs between these life stages may reflect differences in food consumption or utilization. For example, the higher rate and overall volume of food ingestion in the nymph stage, in which planthoppers spend the majority of their time feeding, may activate GST genes to a greater degree compared with adults that spend proportionately more time in other, non-feeding activities such as dispersal and reproduction. It is less clear why GST mRNAs were more highly expressed in eggs than in nymphs but a potential explanation is the rapid metabolism of stored materials during the development of eggs. In *C. riparius*, most GST genes exhibited higher expression levels in eggs compared with male and female adults [45]. Nymphs are less likely to require highly expressed GST genes in order to deal with plant toxins from feeding on unsuitable hosts than are macropterous adults as adult females that are vagile are able to fly from landings on non-host plants in order to select suitable hosts within which to oviposit.

The induction of *S. furcifera* GST mRNAs was similar to that of *L. striatellus*, in which the mRNA of five genes (*LsGSTe1*, *m*, *o1*, *s2* and *s3*) were induced by the four insecticides used in the current study [18]. For example, all GST genes from *S. furcifera* (except for *SfGSTz1*) and four genes from *L. striatellus* (*LsGSTe1*, *o1*, *s2* and *s3*) were highly expressed at 6 hours following exposure to the organophosphate chlorpyrifos (Figure 9). The consistency of toxin induction and ‘time-space’ expression of homologous genes in *N. lugens* and *L. striatellus* is most likely driven by consistencies in environmental pressures. Evidence for this hypothesis lies in the amino acid level of the three homologous genes *LsGSTd1*, *NlGSTd1* and *SfGSTd1*, with the former two having the highest level of similarity (93.6%) (Table 1). Notably, amongst Epsilon sub class genes, *LsGSTe1* and *SfGSTe1* exhibited the highest level of similarity (96.3%), reflecting a similar induction style and expression pattern in *L. striatellus* and *S. furcifera*.

*NlGSTe1* appears to be more important in chlorpyrifos tolerance than *NlGSTm2.* It is unclear, however, whether this sensitivity was mediated by these genes directly, through decreased metabolic activity, or indirectly, through systematic alteration of physiological responses.

Microsomal class GST genes are involved in protecting the cell from oxidative damage and xenobiotics by catalyzing reactions involving a multitude of substrates [52]. Reflecting this important biological role, longevity of MGGST *D. melanogaster* null mutants was significantly reduced compared to wild type in *D. melanogaster* [53]. In the current study, however, *NlGSTm2* knockdown did not have any effect on the mortality of *N. lugens* 3rd instar nymphs. Future studies should extend planthopper observation times in order to determine the effect of *NlGSTm2* knockdown on the longevity of *N. lugens.*

Organophosphate insecticides such as chlorpyrifos, though now banned from use in urban environments and on ornamental plants in some countries, are still applied widely for the control of rice insect pests in China. Resistance to this type of toxin was suggested to be associated with the improved esterases protein level in *N. lugens* [54]. In *A. cracens*, the protein expressed by the Omega subclass gene *AcGSTO1-1* bound the organophosphate temephos [29]. Our findings offer new information on organophosphate insecticide tolerance conferred by the Epsilon subclass and microsomal class genes. In the case of the synthetic pyrethroid pesticide beta-cypermethrin, sensitivity was not significantly increased by either of the silenced GSTs (Figure 11), even though silencing of either *GSTe7* or *GSTe2* by RNA interference in *A. aegypti* resulted in an increased susceptibility to the pyrethroid deltamethrin [55].

## Conclusion

In the present study, the entire GST gene superfamily was identified from the planthopper insect *N. lugens*, an important rice pest in Asian countries. Nine GST genes were found in a closely related and economically important rice planthopper *S. furcifera*. Using phylogenetic analysis and amino acid identity comparisons, the evolution of this gene family was studied. The findings provide a greater understanding of the roles of these genes and gene products in modulating pest-host relationships and could lead to more sustainable insecticidal control and the development of novel ways of manipulating the insect pest-crop relationship. Future work will need to focus on functional studies of the metabolic roles of GST enzymes to different plant allelochemicals in these planthopper species as well as other herbivore species. The profile of gene expression levels of life stages and tissues in this work offers an explanation for the rapid adaptation capacity of migratory (macropterous) *N. lugens* adults. The induced reaction to insecticides differed among the two planthoppers (*N. lugens* and *S. furcifera*) and the previously studied *L. striatellus* and this helps explain former enzymatic level studies, such as the enzymatic activity between different planthopper populations. RNAi provided evidence for involvement of the Epsilon subclass genes and microsomal GSTs in tolerance of *N. lugens* to the organophosphate insecticide chlorpyrifos, although not to beta-cypermethrin. Insecticide resistance is now becoming an urgent problem for the control of the three planthopper members throughout Asia. Our characterization of the GST genes in *N. lugens* and *S. furcifera* sheds light on the genetic basis for this phenomenon, as well as the ability of the planthoppers to overcome the resistance of rice cultivars. More widely, this functional genomics approach can contribute understanding of the role of GSTs in insects at the metabolic level.

## Materials and Methods

### Insects

The strains of *N. lugens* and *S. furcifera* used in all studies, originated from field populations collected close to Hangzhou in eastern China. The insects were reared on susceptible rice seedlings cv. Taichung Native 1 (TN1) (susceptible to almost all herbivores of rice) at 26±1°C, 80% relative humidity, under a 16∶8 hour light∶dark regime for at least 30 generations.

### Sample preparation for different life stages and tissues

For GST gene transcription profiling, *N. lugens* and *S. furcifera* were collected at nine different life-stage, sex and wing-form permutations: eggs, first-instar nymphs, second-instar nymphs, third-instar nymphs, fourth-instar nymphs, fifth-instar nymphs, macropterous adult females, macropterous adult males, brachypterous adult females (except *S. furcifera*) and brachypterous adult males (except *S. furcifera*). For each biological replicate, thirty live individuals of each category were collected and the RNA extracted immediately.

For studies of tissue-specific expression, the midgut, fat body, Malpighian tubule, and remaining body (ie without midgut, fatbody and Malpighian tubule) were dissected from third-instar nymphs. Dissections of the remaining bodies of *S. furcifera* excluded the Malpighian tubules and fat body so that these results could be compared directly with those of *N. lugens*. For each tissue, >300 fresh nymphs were dissected in ice-cold RNAlater (Ambion, Austin, TX, USA) and stored in RNAlater at −80°C until RNA extractions were carried out. All sample tubes were treated with DEPC to protect the RNA from RNase. RNA was extracted using TRIzol reagent (Invitrogen, CA, USA) according to the manufacturer's protocol.

### Identification of GST cDNAs and genomic sequences

Previously, one Expressed Sequence Tag dataset and one transcriptome dataset had been built for *N. lugens* and submitted to NCBI (http://www.ncbi.nlm.nih.gov/) [56], [57]. A second *N. lugens* transcriptome dataset was constructed in our laboratory using a mixture of whole bodies at all developmental stages [58]. Two transcriptome datasets for *S. furcifera* had been built by Xu [59] and a third one was completed in our laboratory (unpublished data). GST genes were identified by searching the sequences in these transcriptome databases for keywords (GST, glutathione transferase and glutathione-s-transferase) or by using the basic local alignment search tool (BLAST) algorithm to search for other known insect GST genes. To confirm the identity of a GST gene, further searches of putative GST cDNAs were conducted using BLASTX to compare the sequence against the non-redundant database at NCBI. If sequences did not have a complete open reading frame (ORF) they were again compared to the cDNA datasets.

Known insect GST genes in GenBank were used to search for similar genes in the *N. lugens* genome sequence (TBLASTN searches against the raw traces and the first genome). Identified genomic scaffold sequences were used to construct GST genes manually using known GST exons as templates and using programs to edit and manipulate sequences (Bioedit, IBIS Biosciences, Carlsbad, CA) and to predict exon-intron splice sites (SplicePredictor, http://deepc2.psi.iastate.edu/cgi-bin/sp.cgi/). Predicted protein sequences were further compared individually with GST proteins from global protein databases to confirm their identity as GST genes using BLASTP in NCBI.

### RNA isolation, cDNA synthesis and full length cDNA clone

RNA was extracted from 20 individuals of *N. lugens* and *S. furcifera* using TRIzol reagent (Invitrogen, CA, USA) according to the manufacturer's protocol. The RNA was treated with DNase (Takara, Japan) to remove any contaminating genomic DNA. The RNA was then reverse transcribed to produce cDNA using the PrimeScript 1st strand cDNA synthesis kit (Takara, Japan). Based on the DNA sequence data obtained from transcriptome searching, eighteen pairs of gene-specific primers were designed and synthesized for the GST cDNAs with complete ORFs (Table 1). The PCR conditions were determined empirically for the amplification of each GST cDNA. Electrophoresis was then carried out using the PCR products. DNA bands of the expected size were excised from the agarose gel and purified using a DNA gel extraction kit (Axygen, USA). These PCR products were cloned into pMD18-T vector (Takara, Japan) and then at least three independent clones were sequenced from each cDNA (GenScript Biotech., Nanjing, China).

### Phylogenetic analysis

Deduced amino acids of GST genes from different insects were aligned using ClustalW (Version 1.83). The percentages of the amino acid identity of different GST genes were determined using DNASTAR software. Phylogenetic trees were determined by the neighbor-joining method with 1000 bootstrap re-sampling statistics implemented in MEGA 4.0. Aligned sequences were then edited and converted into figures with the GenDoc software. The *N. lugens* genome was screened for genomic sequences of the identified GST cDNA sequences above using the BLASTN algorithm. GST cDNAs were matched to these genomic sequences to identify their exon, intron, 5′UTR and 3′UTR with the online tool Exon-Intron Graphic Maker (http://wormweb.org/exonintron). The maximum likelihood method *ω* ratios of the normalized non-synonymous substitution rate (*d*_N_) to the normalized synonymous substitution rate (*d*_S_) were tested with the online tool PAL2NAL (http://www.bork.embl.de/pal2nal/). The GSH and substrate binding sites were analyzed by using the NCBI CD-search program then manually converted into a figure.

### Insecticide treatments

GST genes transcription in response to insecticides were investigated by treating batches of >30 *N. lugens* and *S. furcifera* nymphs to four synthetic insecticides for 6, 12, 24 or 48 hours using microtopical application [60]. Sub-lethal doses of the insecticides (LD_20_ in 24 hours) were used for each insecticide treatment. Third instar-nymphs (1 day after molting from the second instar-nymph stage) were used to avoid any bias from molting during insecticide exposure. At each time point, fifteen insects were collected from each replicate. The nymphs were exposed to: chlorpyrifos (an organophosphate), imidacloprid (a neonicotinoid), buprofezin (an insect growth regulator) or beta-cypermethrin (a synthetic pyrethroid) (Xinnong Chemical Industrial Group Co. Ltd., Taizhou, Zhejiang, China).

### Quantitative PCR of GST mRNAs

RNA was extracted according to the protocols mentioned above. The concentration of each RNA sample was adjusted to 1 µg/µl with nuclease-free water and the RNA was reverse transcribed in a 20 µl reaction system using the AMV RNA PCR Kit (TaKaRa, Japan). The sequences of the specific primer sets for qRT-PCR and the internal genes and primers of *N. lugens* and *S. furcifera* are listed in Table S1. The qRT-PCR was performed on an ABI 7300 Real-Time PCR System (Applied Biosystems, Branchburg, NJ) using the SYBR Premix Ex Taq Kit (TaKaRa, Japan) according to the manufacturer's protocol. A pre-run test was carried out to confirm the constant expression of the actin gene in each sample. After the qRT-PCR assay, the results (threshold cycle value) were normalized to the expression level of the constitutive actin gene. A no template control sample (nuclease free water) was included in the experiment to detect contamination and to determine the degree of dimer formation. A relative quantitative method (ΔΔCt) was used to evaluate the quantitative variation [61]. Data were converted by SPSS v19.0 software and the Mann-Whitney test with a *P*-value of <0.05 was applied for analyzing the fold change of gene expression and its significance.

### Double strand RNA assembly and injection

The *N. lugens* GST gene sequences were amplified by PCR using specific primers conjugated with the T7 RNA polymerase promoter (Table S1). The PCR products were used as templates for dsRNAs synthesis using the MEGA script T7 kit (Ambion, Austin, TX, USA) according to manufacturer instructions. After synthesis, the dsRNAs were isopropanol precipitated, dissolved in ultra-pure water, quantified with Nanodrop 2000 (Wilmington, DE, USA) and their purity and integrity determined by agarose gel electrophoresis. Green fluorescent protein (GFP) dsRNA was produced as control. DsRNAs were injected into live insects through the junction of the prothorax and mesothorax [62]. 20 nl of GFP dsRNA solution or purified dsRNA (5 mg/ml) was injected using a TransferMan NK 2 micromanipulator (Eppendorf, Westbury, NY, USA). For each gene, at least 400 fourth instar-nymphs were injected.

### Quantitative PCR of GST RNA interference sample and bioassay

After injection, nymphs were placed on rice seedlings. Dead individuals were recorded and mortality calculated daily. Thirty individuals were collected for each dsRNA injected group at 36 hours after the injection. Whole bodies of these planthoppers were homogenized with 1 ml homogenization buffer (0.1 M sodium phosphate buffer, pH 7.6, containing 1 mM editic acid, 1 mM 1,4-dithio- threitol, 1 mM phenylthiourea, and 1 mM phenylmethyl sulfonyl fluoride). After centrifugation at 10000×g, 4°C, for 20 minutes, the supernatant was recentrifuged at 20000×g, 4°C, for 40 minutes. The supernatant was then used as the enzyme resource for analysis of the activity of GST activity using a GST activity assay kit (Nanjing Jiancheng Bioengineering Institute, Nanjing, China) [63]. Fifteen insects were collected at 24, 48 and 72 hours following injection and stored at −80°C. Quantitative PCRs for GST mRNA were carried out for each sample according to the protocol above for quantifying the efficiency of RNAi. Sixty 4th instar nymphs were selected at 24 hours following injection, exposed to the insecticides chlorpyrifos or beta-cypermethrin for 24 hours by microtopical application after which mortalities were calculated.

## Supporting Information

Table S1 Primers for gene clone, Realtime PCR and RNA interference. This table shows the primers for GST gene amplification, GST gene Realtime PCR, dsRNA assembling and Realtime PCR assay.
(DOC)

Table S2 GSH and substrate binding sites of three rice planthoppers. *Nilaparvata lugens* (Nl prefix), *Sogatella furcifera* (Sf prefix) *Laodelphax striatellus* (Ls prefix).
(XLS)

Table S3 Intron and Exon length for each of the GST genes in the *Nilaparvata lugens* (Nl prefix) genome.
(XLS)

Table S4 GST amino acid sequences from twelve insect species used in phylogenetic analysis. *Anopheles gambiae* (Ag prefix), *Apis mellifera* (Am prefix), *Bombyx mori* (Bm prefix), *Drosophila melanogaster* (Dm prefix), *Sogatella furcifera* (Sf prefix), *Nasonia vitripennis* (Nv prefix), *Locusta migratoria manilensis* (Lm prefix), *Acyrthosiphon pisum* (Ap prefix), *Toxoptera citricida* (Tc prefix), *Lygus lineolaris* (Ll prefix), *Nilaparvata lugens* (Nl prefix) and *Laodelphax striatellus* (Ls prefix).
(DOC)

Figure S1 **Effect of dsRNA on GST enzyme activity.** Results of GST enzyme activity are expressed as µmol (GSH) min^−1^ mg^−1^ protein. CK, nymph without any injection; GFP, nymph injected with dsRNA of Green fluorescent protein (GFP) gene; *NlGSTe1*, nymph injected with dsRNA of *NlGSTe1*; *NlGSTm2*, nymph injected with dsRNA of *NlGSTm2*. Values with different letters are significantly different as determined using a one-way ANOVA (Duncan's multiple range test, *P*<0.05).
(TIF)

Figure S2 **Survival rate of** ***Nilaparvata lugens*** **after dsRNA injection.** CK, nymph without any injection; GFP, nymph injected with dsRNA of Green fluorescent protein (GFP) gene; *NlGSTe1*, nymph injected with dsRNA of *NlGSTe1*; *NlGSTm2*, nymph injected with dsRNA of *NlGSTm2*. Values with different letters are significantly different as determined using a one-way ANOVA (Duncan's multiple range test, *P*<0.05).
(TIF)
