# Supplementary material for: Genomic Insights into the Glutathione S-Transferase Gene Family of Two Rice Planthoppers, Nilaparvata lugens (Stål) and Sogatella furcifera (Horváth) (Hemiptera: Delphacidae)
Source: PLoS One. 2013 Feb 14;8(2):e56604. doi: 10.1371/journal.pone.0056604 (PMC3572974; doi:10.1371/journal.pone.0056604)
Supplement: Table S1 — Primers for gene clone, Realtime PCR and RNA interference. This table shows the primers for GST gene amplification, GST gene Realtime PCR, dsRNA assembling and Realtime PCR assay. (DOC) [file pone.0056604.s001.doc]

| Table S1 Primers for gene clone, Realtime PCR and RNA interference. | | | | |
| --- | --- | --- | --- | --- |
| GenBank | Gene | Primers | Sequence (5'-3') | cDNA (bp) |
| JQ917467 | *NlGSTd2* | F | CAGTTCTTGTGTGTCGACAA | 832 |
|  |  | R | GTATCAAGTCATGGATCTTCA |
|  |  | RT-F | AGAAGCGCGCTAAGGTCAAC |  |
|  |  | RT-R | GGTGCTCCACCAAACATGTG |  |
| JQ917468 | *NlGSTs1* | F | GAACGTTTTAAGCATTAGTCCTA | 824 |
|  |  | R | GGAAGAGAATCCAGACTTCAT |
|  |  | RT-F | CAGATTTGAAAGGGAGCAATGG |  |
|  |  | RT-R | GCAACTGATTGCCACACTGACT |  |
| JQ917469 | *NlGSTd1* | F | GGTGTGCTTATCTGCAGAA | 790 |
|  |  | R | CGATAAAATGTGTACGGTCTCA |
|  |  | RT-F | GGTGTGCTGCCTCCATTTCT |  |
|  |  | RT-R | GGCAGCTCGGTTTTACACTTCT |  |
| JQ917470 | *NlGSTe1* | F | CCAGTCTAGAGAGGAGACTA | 812 |
|  |  | R | TGTAGGCCCACACTAACAA |
|  |  | RT-F | CGGCGCTCAATTTGGAAGTA |  |
|  |  | RT-R | GGGATTGTGTGTTGAGGATTCA |  |
| JQ917471 | *NlGSTo1* | F | CCATATCAGATCGTAACTGAA | 911 |
|  |  | R | CACTATACACCCACCTTTGT |
|  |  | RT-F | GAAGCTCGATTCCCCTGTTTG |  |
|  |  | RT-R | AATGATGGGCGTGCTGATCT |  |
| JQ917472 | *NlGSTt1* | F | GTCAAAAAATTACTTTAAA | 1120 |
|  |  | R | GCTAGGTCCTAGACTTTAAA |
|  |  | RT-F | AGCAAGCCAAAGTCGACGAA |  |
|  |  | RT-R | GTGACCATCGGCATCAGAAA |  |
| JQ917473 | *NlGSTm2* | F | CTGTTTCTCAGTTGTCAGAA | 638 |
|  |  | R | CAGAGACTGAACAGTATAAGT |
|  |  | RT-F | ACATTGCTACCAACCCCTCCTA |  |
|  |  | RT-R | ATGACCACGATCGCGTACAC |  |
| JQ917474 | *NlGSTs2* | F | GGAAGATAGCTGAGAGAACA | 704 |
|  |  | R | GCTTTAGCACTAATATTCTAGTT |
|  |  | RT-F | GAGCAATGGCCAACAATCAA |  |
|  |  | RT-R | ACCTTGCAATGGCAGTCGAT |  |
| JQ917475 | *NlGSTm1* | F | CTTTATTAACAATAAACACGCTT | 560 |
|  |  | R | CACTCTAGTCAAGATTGTTCAA |
|  |  | RT-F | GCTGCCAAAGTCTGTGGTCAA |  |
|  |  | RT-R | GCTGCCAAAGTCTGTGGTCAA |  |
|  | *NlGSTs3* | F | ATGGCTCCATCTCGCTAC | 141 |
|  |  | R | GTAGCGAGATGGAGCCAT |
|  | *NlGSTz1* | F | GATACAGAAATCTGTGAGCA | 700 |
|  |  | R | AGCAGTACACACAGCATCGTT |

| Table S1 Primers for gene clone, Realtime PCR and RNA interference. | | | | |
| --- | --- | --- | --- | --- |
| GenBank | Gene | Primers | Sequence (5'-3') | cDNA (bp) |
| JQ917476 | *SfGSTm1* | F | CTACTCTAGGCCTTTTGTTGTA | 540 |
|  |  | R | CAAAACTCTATTGGAACGATAGT |
|  |  | RT-F | GACGAGCGCATCTGAATGATC |  |
|  |  | RT-R | AGATTCAGCGCCAGCCAATA |  |
| JQ917477 | *SfGSTt1* | F | CGTAGTTTTTCAACAAGTGGTT | 844 |
|  |  | R | GCCACTCTCCACAAGACAAATA |
|  |  | RT-F | GAGCATCTTCAACCCGAATTTG |  |
|  |  | RT-R | TGAGGATCGCCACACTTTCA |  |
| JQ917478 | *SfGSTm2* | F | GGCACGTCGCACGTAAAAAT | 567 |
|  |  | R | TATGAGTCATTTTTGACCAAG |
|  |  | RT-F | CTGCTCTACATCGCCACCAA |  |
|  |  | RT-R | TACCTTTGTGTACGCTGTGG |  |
| JQ917479 | *SfGSTo1* | F | CAAGTGAAGATTTCCTGCTACT | 779 |
|  |  | R | GATACTGCAATTGATACGACAA |
|  |  | RT-F | TCCGTACGACCCAGTGTACATC |  |
|  |  | RT-R | TGTGCCTTCCACCACAAGAG |  |
| JQ917480 | *SfGSTs1* | F | GCACCTCGACTGTTCAACAA | 795 |
|  |  | R | GAAGAATCAAGTGCCAGACTT |
|  |  | RT-F | AGTGCCTGTTTTGGAAATCGA |  |
|  |  | RT-R | CCATTCGTCCTTTCCAGCAA |  |
| JQ917481 | *SfGSTd1* | F | CTCAATAACTCACTCCACTGTT | 772 |
|  |  | R | GATAGAATTTGTACGGTCTCAA |
|  |  | RT-F | CTCAATAACTCACTCCACTGTT |  |
|  |  | RT-R | GATAGAATTTGTACGGTCTCAA |  |
| JQ917482 | *SfGSTe1* | F | CAGAGAATACGCAGTCTGGAT | 898 |
|  |  | R | CTAGAAAATAGAGACAGTCTTA |
|  |  | RT-F | AAATCTTTTCCGGCGTTGAA |  |
|  |  | RT-R | ACTCCTTGTTGATTGGCCGTAT |  |
| JQ917483 | *SfGSTd2* | F | GTGCCTTTGGTTACTTATCAGTT | 759 |
|  |  | R | GCCACACATTGAACAGTGTGA |
|  |  | RT-F | TTTTGAACGAGAGCCGTGCCA |  |
|  |  | RT-R | AAGAAGCGCGCCAAGGTCAA |  |
| JQ917484 | *SfGSTz1* | F | CAGTATTACTCCTGTAGCTGAT | 783 |
|  |  | R | GACAGTCTGCTGGAATCAGAA |
|  |  | RT-F | GTGTGCTGGTAAATATTGTG |  |
|  |  | RT-R | GGTTCCACGTAGATCTGCGA |  |

| Table S1 Primers for gene clone, Realtime PCR and RNA interference. | | | | |
| --- | --- | --- | --- | --- |
| GenBank | Gene | Primers | Sequence (5'-3') | length (bp) |
| JQ917470 | *NLGSTe1* | RNAiF | ggatccTAATACGACTCACTATAGGGAG | 615 |
|  |  |  | ATGACAATCGACTTCTACTACATG |
|  |  | RNAiR | ggatccTAATACGACTCACTATAGGGAG |  |
|  |  |  | AGTCAACTCCTTGCTGATTGG |  |
|  |  | RT-F | CAGCGTCATACACAGCTTTTCTG |  |
|  |  | RT-R | CGCCGCCAAACATAAATTG |
|  |  |  |  |  |
| JQ917473 | *NLGSTm2* | RNAiF | ggatccTAATACGACTCACTATAGGGAGA | 450 |
|  |  |  | ATGTCTTCAAGTTTGTACACCAC |
|  |  | RNAiR | ggatccTAATACGACTCACTATAGGGAGA |  |
|  |  |  | GAGACTGAACAGTACAACTTGC |  |
|  |  | RT-F | AAGCTACACGTGTTCAGGAACGT |  |
|  |  | RT-R | TATCCGTGGTGTACAAACTTGAAGA |
