# Supplementary material for: Genomic Insights into the Glutathione S-Transferase Gene Family of Two Rice Planthoppers, Nilaparvata lugens (Stål) and Sogatella furcifera (Horváth) (Hemiptera: Delphacidae)
Source: PLoS One. 2013 Feb 14;8(2):e56604. doi: 10.1371/journal.pone.0056604 (PMC3572974; doi:10.1371/journal.pone.0056604)
Supplement: Table S4 — GST amino acid sequences from twelve insect species used in phylogenetic analysis. Anopheles gambiae (Ag prefix), Apis mellifera (Am prefix), Bombyx mori (Bm prefix), Drosophila melanogaster (Dm prefix), Sogatella furcifera (Sf prefix), Nasonia vitripennis (Nv prefix), Locusta migratoria manilensis (Lm prefix), Acyrthosiphon pisum (Ap prefix), Toxoptera citricida (Tc prefix), Lygus lineolaris (Ll prefix), Nilaparvata lugens (Nl prefix) and Laodelphax striatellus (Ls prefix). (DOC) [file pone.0056604.s004.doc]

**Table S4 GST amino acide sequences from twelve insect species used in Phylogenetic analysis.**

>AgGSTd1-XP_313050

MDFYYLPGSAPCRAVQMTAAAVGVELNLKLTDLMKGEHMKPEFLKLNPQHCIPTLVDNGFALWESRAIQIYLAEKYGKDDKLYPKDPQKRAVVNQRLYFDMGTLYQRFADYHYPQIFAKQPANPENEKKMKDAVGFLNTFLEGQEYAAGNDLTIADLSLAATIATYEVAGFDFAPYPNVAAWFARCKANAPGYALNQAGADEFKAKFLS

>AgGSTd10-AAM61894MELYYNIVSPPCQSVLLVGKKLGITFDLKEVNPHLPEVREQLRKFNPQHTIPTFIEDGHVIWESYAIAIYLVEKYGNGDDALYPRGPKVRSVVNQRLFFDNGLMFKSAIEYVECILKKKLEPTEEMQQRLKKALGLLESFVKERAFVASDHLTIADICLLSSVTLLTGIKYDLAAFPGITAWVARVTGELPDYGEFHKELYEKSMEYIKTL

>AgGSTd11-AAM53609

MDFYHLPLSAPCQSIRLLAKALGLHLNLKEVDLLKGEHLKPEFLKINPQHTVPTLVDNDFVLWESRAILTYLCEKYGKNDGLYPKDPKKRAVVNQRLYFDMGTLYQRFSQAFYPVMMEGKELNPELVVKLDEALEFLESFLDKTPFAAGDKLTVADFSLLTSITTIDVTAGHDLSKYANIQRWYSQLQESVAGHQDICVEGAIQFRDSFNPINK

>AgGSTd12-XP_313666

MDLYYHIRSPPCQPVVFLARHLGLEFNHIVTSIYDPADFEVLKKVNPQHTIPTLVDNGHILWESYAILIYLAEKYALDDSLYPKDVCERSIVHQRLFFDSGMFQNTTLQAVLSHLRNNPITDEHLAKVKRGVEIVEMYLTDSPYVAGQKLTIADFSIFVSFCSLDMMKYDLTAYPNVQRWFAKMGTHIPDLEPTRKTIEEELRALLQSMNK

>AgGSTd2-CAA96104

MLDFYYLPGSAPCRAVQMVAEAVHVKLNLKYLDLMAGAHRSPQFKLNPQRTIPTLVDGSLVLSESRAALIYLCDQYGDEDNDWYPRDTIQRAIVNQRLFFDACVLYPRFADFYHPQVFGNAAPDGRKRLAFEKAVELLNIFLSEHEFVAGSKMTIADISLFATLATACTLGFILRPYVHVDRWYVTMVASCPGAQANVSGAKEFLTYK

>AgGSTd3-AAC79992

MDFYYLPGSAPCRAVQMTAAAVGVELNLKLTDLMKGEHMKPEFLKLNPQHCIPTLVDEDGFVLWESRAIQIYLVEKYCAHDPALAERLYPGDPRRRAVVHQRLFFDVAILYQRFAEYYYPQIFGKKVAGDPDRLRSMEQALEFLNTFLEGERFVAGGDDPTIADFSILASIATFDAAGYDLRRYENIHRWYEQTGNIAPAADKNLAGAKIFGLYFRQK

>AgGSTd4-AAM53607

MDYYCNFVSPPSQSVILVAKKLGIKLNLRKINIYDPVAMDTLSKLNPHHILPMLVDNGTVVFEPCAIVLYLVEMYAKNDALYPKDALVRCVVNQRLFFDVSTLFKQIYENVHVQMRNSQPSEKQVQRLQKAVDVLESFLYERSYTAADQLTVADICLLVTVNALTLWLGYELAPYPRIRDWLGRVVAEIPGCAEFQREVEDATRAYVVNRKI

>AgGSTd5-AAM53606

MELYSDIVSPSCQNVLLVAKKLGIALNIKKTNIMDATDVAELTKVNPQHLIPTFVEDDGVIWESYAIAIYLVEKYGQDDALYPKDPKVRSIVNQRLFFDIGTLYKNILANVDVLIEKQQPSAELRGKLEQALDLTEKFVTECRFVAADHLTLADIFMLGSITALEWFRYDLERYPGIRGWVERVTAQFPDYSNFHKEIREATKQYVATHCPHLEY

>AgGSTd6-AAM53608

MPRLDLYYNIISPPCRVVLLFAKWLKLELNLIELDVLKRDHYKPEFLKLNPQHYIPTLVDADGDVVVWESSAILIYLAERYGAADDDTLYPKDIALRAKVNQRLFYDIGTLMRSVTTYYHPILMGGEGKLEDFKKVQDAVGVLDSFLSASRWTAGDHITVADFAIAVTVAALDGLLNFDFSVYPNVHRWYEQCKRELVGYTDITKEAAQRTQAFLERFRAMR

>AgGSTd7-XP_562676

MTPVLYYLPPSPPCRSVLLLAKMIGVELELKALNVMEGEQLKPDFVELNPQHCIPTLDDHGLVLWESRVILAYLVSAYGKDENLYPKDFRSRAIVDQRLHFDLGTLYQRVVDYYFPTIQLGAHLDQTKKAKLAEALGWFEAMLKQYQWSAANHFTIADIALCVTVSQIEAFQFDLHPYPRVRAWLQKCKDELQGHGYKEINETGAETLAGLFRSKLKQ

>AgGSTd8-AAG45165

MDFYYHPASPYCRSVMLVAKALKLSLNLQFVDLMKDEQLRPTFTVLNPFHCVPTLVDNDLTMWESRAILVYLVDKYGRTNSRLYPKDAKTRAIINQRLFFDHGTLGTRLEDYYYPLYFEGATPGGEKLEKLEEALAVLNGYLINNPYAAGPNITLADYSLVSTVTSLEVVQHDLSKYPAISAWYEGCKATMADFQEINESGMQQYRLTSSLVPHLQLLHMPFAE

>AgGSTd9-XP_313058

MDLYYNILSPPSRAILLLGEALQLKFNLISLDVHRKDYVNPAFKKINPQHTVPTLVVDGVAICEPGAILIYLAEQYAPAGTTYYPPDPLRRAIVNQRLLFECGTLYKCIFVYYSPVVLERATPVETDRQKLIEAVAVLDGILQHSAFVAGDCLTVADYSLVCTVSMLVVLKFELAPYAAVRRWYERCKEVIAGYTDLTQRAVTMFQKWMEQENSKG

>AgGSTe1-XP_319969

MPKPVLYTVHLSPPCRAVELTAKALGLELERKLVNLLAGENLTPEFLKLNPKHTIPVLDDNGTIISESHAIMIYLVRKYGQGEGKDALYPTDIVEQARVNEALHFESGVLFARLRFITELVFFARKPEIPEDRIEYVRTAYRLLEDSLQSDYVAGSRMTIADLSCISSVASMVGFIPMERSEFPRVHGWIERMKQLPYYEEINGAGATELAEFIVDMLAKNAKL

>AgGSTe2-AAG45164

MSNLVLYTLHLSPPCRAVELTAKALGLELEQKTINLLTGDHLKPEFVKLNPQHTIPVLDDNGTIITESHAIMIYLVTKYGKDDSLYPKDPVKQARVNSALHFESGVLFARMRFNFERILFFGKSDIPEDRVEYVQKSYELLEDTLVDDFVAGPTMTIADFSCISTISSIMGVVPLEQSKHPRIYAWIDRLKQLPYYEEANGGGGTDLGKFVLAKKEENAKA

>AgGSTe3-XP_319972

MAPIVLYSTRRTPAGRAVELTAKMIGIELDVQYIDLAKKENMTEEYLKMNPMHTVPTVNDNGVPLYDSHAIINYLVQKYAKDDTLYPAKDLVKQANINALLHFESGVLFARLRWILEPVFYWGQTEVPQEKIDSVHKAYDLLEATLKTSGTDYLVGGTITLADISVSTSLCTLNALFPADASKYPLVLAYLKRLEQTMPHYQEINTDRANDALQLYNQKLGKV

>AgGSTe4-XP_319967

MPNIKLYTAKLSPPGRSVELTAKALGLELDIVPINLLAQEHLTEAFRKLNPQHTIPLIDDNGTIVWDSHAINVYLVSKYGKPEGDSLYPSDVVQRAKVNAALHFDSGVLFARFRFYLEPILYYGATETPQEKIDNLYRAYELLNDTLVDEYIVGNEMTLADLSCIASIASMHAIFPIDAGKYPRLAGWVKRLAKLPYYEATNRAGAEELAQLYRAKLEQNRTNAK

>AgGSTe5-XP_319966

MATNPIIKLYTAKLSPPGRAVELTAKLLGLSLDIVPINLLAGDHRTDEFLRLNPQHTIPVIDDGGVIVRDSHAIIIYLVQKYGKDGQTLYPEDPIARAKVNAGLHFDSGVLFSRLRFYFEPILYEGSAEVPQDKIDYMKKGYELLNDALVEDYIAGSSLTLADVSCIATIATMEEFFPMDRSRYPALVAWIERLSRTLPEYDQLNQEGAVEFAEICESLRLKNGASVAAK

>AgGSTe6-AAL59655

MSSKPVLYTHTISPAGRAVELTVKALNLDVDVREMNVFKGQHMSDEFKKLNPVQTIPTLDDNGFVLWDSHAIMIYLARRYGADSGLYTDEYEQQARINAALFFESSILFARLRFCTDNLTVLGKSAIPEENLQRALEGLQRLERMLQSEYVAGDQLTIADLSCVSSVATLHLMLKPSAEEFPKTFAWMDRLSKLPYYGEVMGRGLKAAGELMQTLGSKNSGGGGDGN

>AgGSTe7-AAM61879

MEPSRLVLYTNRKSPPCRAVKLTARALGIELVEKEMTLLRGDKLMEEFLKVNPQQTIPVLDDGGIVITASHAITIYLVCKYGRDDGLYPSELVRRARVHTALHLEAGVIFSRLSFLFEPVIYSGKSYFHSDRIEHIRKAYRLLEDSLVDQYMVGESLTIADFSCISSIATLVGVVPLDESKFPKSTAWMRRMQELPYYEEANGTGALELAEFVLGKKEANASQFL

>AgGSTo1-XP_315763

MSNGKHLAKGSSPPSLPDDGKLRLYSMRFCPYAQRVHLMLDAKKIPYHAIYINLSEKPEWYLEKNPLGKVPALEIPGKEGVTLYESLVLSDYIEEAYSAQQRKLYPADPFSKAQDRILIERFAGSVIGPYYRILFAADGIPPGAITEFGAGLDIFEKELKARGTPYFGGDKPGMIDYMIWPWCERVDLLKFALGDKYELDKERFGKLLQWRELMEKDDAVKQSFISTEDHTKFLQSRKNGENNYDILA

>AgGSTs1-AAM53611

FNVKALGEPLRFLLSYGNLPFDDVRITREEWPALKPTMPMGQMPVLEVDGKKVHQSVAMSRYLANQVGLAGADDWENLMIDTVVDTVNDFRLKIAVVSYEPDDEIKEKKLVTLNNEVIPFYLEKLDDIARDNNGYLANSKLSWADIYFTAILDYLNYMTKSDLVANHPNLQRVVDNVTSIESIRSWIDKRPKTEI

>AgGSTt1-XP_311299

MSKNLKYYYDLMSQPSRALWIFLEKTKLPYEKCLINLGKGEHLTEEFKAINRFQKVPCITDSQIKLAESVAIFRYLCREYQVPDHWYPADSRRQALVDEYLEWQHHNTRATCAIYFQYVWLRPRMFGTKVDPKQAEKYRGQMEGTLDFIEREYLGSGARFIAGDEITVADLLAACEIEQPRMAGYDPCEGRPNLTQWMARVRESTNPYYDQAHKLVNKFAQDTASKAKL

>AgGSTt2-XP_316865

MSRSVKLYYDLMSQPSRALYIFLSTNKIPFDRCPIALRKMQHKTDEYRRQVNRYGKVPCIVDGSFRLAESVAIYRYLCREFPTDGHWYPSDTVRQARVDEYLSWQHLNLRADVSLYFFHVWLNPLLGKEPDAGKTERLRRRLDGVLNFFDQELLSAGSGQAFLAGDRISIADLSAACEIEQAKIAGYDPCEGRPALASWLTAVRERTNPYYDEAHKYVYRLSPDHIVTPVVAEDE

>AgGSTu1-XP_309135

MKLYAVSDGPPSLAVRMALEALNIPYEHVSVDYGKAEHLTAEYEKMNPQKEIPVLDDDGFFLSESNAILQYLCEKYAPTSDLYPNDPKDRALVNHRLCFNLAFLYPQISAYVMAPIFFDYERTAIGLKKLHLALAAFETYLQRTGTRYAAGSGLTIADFPLVSSVMCLEAIGFGLGERYPKVQAWYDGFKQAHPSLWAIAAKGMEEIAEFEKNPPDLTGMVHPIHPIRKPAAK

>AgGSTu2-XP_307765

MPAPTLYYFPMSPPARAVLLLMKELELPMNLKEVNPLAGETRTEEFMRMNPEHTIPTLDDNGFYLGESRAILSYLIDAYRPGHTLYPNIPKEKALINRVLHHDLGSFYPKFFGTIGALFSGAATEISDEMKTTTQKALTDLEHYLTRNDYFAGENLTIADLSLVPTIASAVHCGLDLTNYPRLNAWYESCRVLKGFEDDQEAARQVGEYLRSKFPTGLEALN

>AgGSTu3-AAM61891

MAPLILYHFPGSPPSRSALLALRNLDLDAEVKIVNLFAGEHLADEFVAINPDHTVPTLVDEDYILWESKAIVTYLAEQYKPGCTLYPSEPKKRGLINHRLYFDSGTLFVALRNVLMTVLRSGETRIPQEKKDAVYKALEKLDSYLDGCDWIAGEECTLADLCALANVATLKEIGVGMEGYANVSGWYERCRELPGFDENEEGASFLGNAFKSKLEEQF

>AgGSTz1-XP_312009

MANVDILPESQPILYSYWRSSCSWRVRIALNLKEIPYDIKPISLIKSGGEQHCNEYREVNPMEQVPALQIDGHTLIESVSIMYYLEETRPQRPLMPQDVLKRAKVREICEVIASGVQPLQNLIVLIHVGEEKKKEWAQHWITRGFRAIEKLLSTSAGKFCVGDEITLADCCLVPQVFNARRFHVDLRPYPIILRIDRELEGHPAFRAAHPSNQPDCPPEAAK

>AmGSTd1-NP.001171499

MPIDFYQLPGSPPCRAVALTAAALDIEMNFKQVNLMNGEHLKPEFLKINPQHTIPTIDDNGFRLWESRAIMTYLADQYGKNDTLYPKDLKKRAIVNQRLYFDMCSLYKSFMDYYYPIIFMKAVKDQAKYENIGTALSFLDKFLEGENYVAGKNMTLADLSIVSTVSTLEALDYDLSKYKNVTRWFAKIKPEIPKYEEYNNAGLKMFKELVNEKLSKK

>AmGSTo1-XP.624501

MSSKHLTIGSVAPPIVPGKIRLYSMRFCPYAQRIHLVLDAKHIPHDVVYVNLTHKPDWLLEKSPLGKVPCIELEGGEILYESLVIAEYLDDTYPQNKLYPNDPLARAKDKLLIGRFNSVINTMCKLFINTSIDQDIFDEALSELELFERELASRGTPFFHGNSPGMLDFMIWPWWERSNTIKMLRGDQFTIPHDRFKRLLEWRSAMKENPAIRSNYLDTEIHAKYMQSRRAGTPQYDLITD

>AmGSTs1-NP.001153742

MSTYKLIYFNIPGLAEPIRFLLHQSGIKFEDKRIDIEEWPKIKSEMPLGQVPILEIDGKVYYQSKAISRLIAKRNNFYGSNDEEAFLVDATVETIDDLRQPITQHYWEKDHAFKAKLKINVDTKVPLLLNKLEEQVKKNKGYFVNGKLTWADLFYAAIEESLTDMLGYDLNKDHPELKKLSEKIKSLPNIKTYLKNRPKSMVIP

>AmGSTs4-NP.001136128

MASYKLVYFNVMGLGEPIRFLLSYGGVEFEDVRITHSSEDWSNMKPTTPFGQLPTLEINGKVYSQTLPICRYLAKQFNLLGKTDLDTLQIDAIASALYDFRWLTISSYYRESDPVLKAKKKVDVMTRVIPFYLNKLEELAKNNGGYLHGDKLSYADLFFVGISDSLNTAYESDITNDKPYLKSLKQKILAIPNIKAWVEKRPKLEF

>AmGSTt1-XP.624692

MSLKLYYDLLSQPSRTLYIFLKICDIPFEAKLINLAKGEQFISKYQNIHPFQKVPAIEHNGFNMIESVAILRYICREFNVADHWYPKDSKLQLRVDEYLEWHHLNTRLHCSMYFLKKFLIPKLSGQETTVTQENIMKYEKNMIKILDVLENVWLKDKIFLTGSEISIADILAACEVEQVRIVGYNLQENRPRIAAWMKYVENKTSPYYQEAHIFLNKLATKTKQDVKSKI

>ApGSTd-AK340504

MPIDFYYTPGSPPCRSVLLTAKALGLELNLKTLDLHHGEHMKPEFIKLNPQHCVPTLVDGDLVLWESRAIIVYLAQAYGKDDSLLPKDPKKQALVNQRLQFDVSTLYPAFSDQYYPWIFAGVPKSDDKEKKIHDALGFLDIFLGSSTWAAGDSVTVADLALVASISTIEAVGVDLSKYANVSKWFEKCKTTLVGYQEFNQKGIDGFKIMVANLTKK

>ApGSTd-AK341049

MTIDFYYASWSPPCRTVELVAYILKVKLNPIETIPSKGDTQKPEYKQLTPQHTIPTIVDNGFVLSESRAICKYLVEKYGSATGPYSKEQLYPKDLQKRAAIDHRIDFDLGSLYRRASDYFSPVFMTGHFGTAALPKLKAALEILDTYLAKTKWVAGPEVTLADIVVVVTISSLEIVGYELTNYPNILRWFKAAQTTLPGYNEANHKGRIEFKDYLFSKLKNN

>ApGSTo-AK340396

MAIKHLSKDSVEPPKVPGSLRFYSMRFCPYAQRVQLILNAKGMPHDTVFIDLSDKPEWYLKIFPAGKVPALIYDDKFLSESLLLADFLDKQYPEPPLQASSPLQTILDKLVIESFGKVGTAFYKLIMTTKEIEKQNFDELVASLIPIETELVERGTKFFGGNKPNMVDYMIWPWFERLDAINPYSNGTFVIPFEDKFPRLAEWKSLMIADKAVAPYYITPEKHAEHFTKRKAGLPAYDI

>ApGSTs-AK339849

MSYKVTYFNITALAEPIRFLLSYLNIDFEDFRFEREQWPTLKPTMPFGKVPVLEIDGKVLNQSTAITRYLSKKAGLAGSDDWESMLIDIAVDNIHDLRQAIALYAYDSNEATKEARYAPLINETIPFYMDKFEKIVEENNGYFVNGKLSWADLFFVAILDYLNFMAKIDLLEGRPKLKALKEKVLEVPQIKAWVAKRPTDNP

>ApGSTs-AK340693

MTTYKLTYFNLTARAEQIRFLLSYLNVDFEDVRFEREQWPAIKPTMPFGKVPVLEIDGKTFNQSIAICRYLAKKAGLAGDDEWESLLIDVAVDNIYEIRQEIMNYYHEPNEEIKSKLRGPIVNDSIPFYIDRFENIVSENGGYFVNGKLSWPDLYFVSILDHIKSVIDVDLVDGRPHFTAFKHKVLAIPQIKSWIAKRPKSQ

>ApGSTs-AK341181

MAVYKLTYFNFPALAEPIRFLLSYLEIDFEDVRFEREQWPSIKPTMPFGKVPILEIDGKVLNQSAAISRYLSKKAGLAGSDEWESLLIDIAVDNVNDLRQAIALQVFDSNEESKAEKYVTLINETIPLYMNKFENTVVENDGYFVNGKLSLADIHFVAIIDFLSFLAKVDLLEGRPNLQAHKNKIFDIPQIKSWIAKRPAFSMKL

>ApGSTt-AK339936

MVTLKFYYDFLSQPCRTLYIFMKKTKIPFEPKPVNLRQGEHLTKEFVSLNPFKKVPFIDDKGTVLIESVSILRYLCRTYNVADHWYPKDIQRQALVDQYLEWQHNNTRAHCTEYFRHKALWAAKNWPGSKY

>ApGSTt-AK340178

MAKLIYYHNLLSQPSRALYMFFKKAEVPFEGKVVDLLKGEQFTAEFEAINPFKKVPVVNANGFVLIESIAILRYICRTYNVADHWYPKDSVKQAQVDEYLEWQHTNTRADCALYYLHKVLWPVMNGKPVNEQRVAQLEKKMITTLDLIENVWLKNKTFLSGNEISISDIIAICEIDQTRIAGYNPYANRPNLSNWKMRTATYLSPYYEEANEILEMHVAKYNKKYGKLNSHI

>BmGSTd1-AJ006502

MPVQPIKLYYLPPSPPCRAVMMTARVLELDLHLITTNIMNGEHMTPEYLKMNPQHTIPTMDDNGFILWESRAIQTYLVNAYGKDDSLYPKNPRQRAIIDQRLNFDLGTLYLRYLNLYTPILFRGEAYDQEKADKFDEALGWLNTFLDGRPFVAGENMTVADITIVVTITNIDAFGYDFSSHENIAKWFERTKKMLEPYGYDEIDVTGAKMLASFLKKE

>BmGSTd2-AB176691

MTIDLYYVPGSAPCRAVLLTAKALNLNLNLKLVDLHHGEQLKPEYLKLNPQHTVPTLVDDGLSIWESRAIITYLVNKYAKGSSLYPEDPKARALVDQRLYFDIGTLYQRFSDYFYPQVFAGAPADKAKNEKVQEALQLLDKFLEGQKYVAGPNLTVADLSLIASVSSLEASDIDFKKYANVKRWYETVKSTAPGYQEANEKGLEAFKGLVNSMLKK

>BmGSTd3-DQ355374

MAIDLYFTAGSAPCRVVLLVAAALDLQLNLKPLNLWEREQLQADFLKLNPQHTVPTIVDEGFPLWESRAISRYLVNKYGGDSSSLYPKDLMARALVDQRLDFDIGTLYPRFAQYFYPQVFGGAKPDAAALKKLEEALVFLNAFLEGQKYVTGDVLTIADLSLVATISTIDAAEISLKSYPNVEKWFELMKTTAPDYQNANQKGIDEFKKLIAQMKAKTEL

>BmGSTe1-AY192575

MVLTLYKMDASPPVRAVYMVIEALSIPNVKYVDVDLLAEDHLKEEFLKLNPQHTIPMLTDDKFVIWDSHAIATYLLNKYGKGSSYYPEDPEKRALIEMRLHFDSGILYPALRENDEPIFFWGETTFKPEGLAKIKSAYDFTEKFLSDSPWIAGDDVTVADMSCVATIGSLDALLPINEKEYPKITSWLKRCSELDFYQRGNNVKGLLEFKALLKQYLSRGKE

>BmGSTe2-DQ355376

MSIIIYQTSVSPPARSALMVVNILGIKAETREVTLPRRDHYSQEYLEKNPLHTVPILEEDDLVIADSHAIITYLVSKYGEEKHESMYPKDLKIRAITDQMLYFDATILFPRLKTVIYSVVRGQGMSRQQIADIQEAYDVLEIYLSKNIFVAGNEFTVADISCVATLSSLDCVLPVDKKHVNVNRWWATLSNEKWYKEVNVPGLELFRSFIKQFLK

>BmGSTe3-EF506488

MSLMLYKLNASPPARTAMMVCELFKVPVKMVDVNLSKGEHFSPEYLKRNPLHTVPTLEDGDLIITDSHAIAMYLADKYGKDDSLYPKDLKSRAIVNQRLFFDSTVLFSRMRSVTFPVIIEGCKTVTEKQINDIIEAYGYVETYLSNTKFIATNNLTIADISAYAVVSSLLFIVPLDGAKFPKTQTWLNEMEKKPFAQKYNVNGVAELGALLKEKLGS

>BmGSTe4-EF506489

MVFILYKKDTSPPCRSVQMVLHELGIYDVELIEVNLPERDHLKEEFLRMNPQHTVPTLIDGDFIIWDSHAIVTYLVNRYAKNDTLYPKEPKQRAIVDQRLHFDTGVLFAILRATAEPVLYNNEKSFKQENLEKMEAAYEFVEKFLTSDWLAGDQVTLADICCVSTISSMNVIVPIDKKKYPKIISWLQRCSEQEFYKKANEPGLKKFIEMFKNKIGN

>BmGSTe5-EU216542

MVLTLYKLDASPPVRSVYMVIEALKIRDVEYVDVNLLEGSHLKEEFLKMNPQHTIPLLKDDDFLIWDSHAISGYLISVYGADDSLYPNEPKKRALIDQRLHFDSGILFPALRGVAVIIFFNLLCLGQDELIIFRGEKEIRPENLAKIKSAYDFTEKILSSDWIAGDEFSLADICCVTSISTLNEMVPIDGSLYPKLASWLDRSSQLPIYKKANEPGLLQFREIFKNKTS

>BmGSTe6-EU216543

MTPILYKTDASPPARAVMMIVDILGLKVDEQELNPILRQQDTPEFKKKNPMRTIPILEEGDFYLADSHAIMLYLIDKYGKPEHAHLYPSEKRKRATINQRLFFDCGVLFPRLRAVMAPTYAGKLAELNRNMIKNIEDAYSIMESYLTENLYLADEVVTVADISAITTISSLNGLYPVDEKSKWINRMNDKEYCRKINTPGSELHVAGLIALMDNTKHNQQSKL

>BmGSTo1-DQ311183

MSEKHLQTGDVLPPYSGKLRVFAMRFCPYAERTVLTLNAKNIPYDLVFINLDQKPEWIFNFSPKGTVPALEYEPGKALFDSNIINVYLDEKYPEIPLQASDPLRRAQDKILVESFAPAQSAYYTAAFNAQALEPSMVETYHKGLEGLQKELETRSTKYLHGDEPGWVDYTLWPFLERFEALPLIGKAEFAIDQTKYERLVTYIEAMKNVPAVKSYFLAAETHAKFIESRAQGDANYNMLDTSAVCCMRPRKKKE

>BmGSTo2-DQ355373

MSAIKDSRNINFNIKHLRKGDPLPPFNGKLRVYNMRYCPYAQRTILALNAKQIDYEVVNIDLIDKPEWLTTKSAFAKVPAIEIAEDVTIYESLVTVEYLDEVYPKRPLLPQDPLKKALDKIIVEASAPIQSLFIKILKFSDTVNEEHVAAYHKALDFIQEQLKNRGTVFLDGSEPGYADYMIWPWFERLRAFAHDERVRLEPSKYSLLLEYIDNMLKDSAVSQYLIPLEILAKFHEAYTKKERPNYELLNECLKSF

>BmGSTo3-DQ443293

MTYFHSVNAGVIPPPALTDKLRLYHVDMNPYGHRVLLVLEAKRIKYEVYRLDPLRLPEWFRAKNPRLKIPVLEIPTDQGDRFLFESVVICDYLDEKYTRHTLHSHDPYVKAQDRLLIERFNELIKGSLECFDTNFAFGSEQIIQTLEIFEKELTNRGTNYFGGNRPGMLDYMVWPWVERLYLLRCVNDRKFVEKKSLFPNFADWGDQMQLDDIVKKHAHSPQEYFDYYKNARAHSMGYYL

>BmGSTo4-EU216544

MVSPKINFNTKHLGKGDPLPPWSGKLRVYNMRLCPFAQRTILTLNAKQIDYEVINIDLVNKPEWLPTKSIFGKVPTIEVEDGVCICESLIIAEYLEEVYPEIPLISKDPIKKAYEKIIIEASEPIFVMYFKVMRTPDTINDETLMSYHKALTFFEGQLRNRGTRFLGGEKPGFADYMIWPWFERIQSMNDEKLKIKSAKFDLLVAYIENMYKDPAVSQYLLPKDVMDKLHAEYKTGKFEVQSIEDLL

>BmGSTs1-AY297161

MPKVVYHYFACKALGESGRMLLAYGGQDFEDHRVLSADWPDFKPKTPFGQTPVLVIDGKQYAQSTAICRYLGRKYGLAGANDEEAFEIDQNVEFLHDIRAKAAAVYYEADEELKAKKHEDFSKNVYPDMLKKLNSIVEANKGHIAAGKLTWGDFVFTSMFDYLKTMLQIPDLEVQYPAFKKVLQSVLTQPKVKAFLDLGRPYEFEF

>BmGSTs2-AB206971

MPNVKFYYFPVKALGESQRLLLAYGGQEFEDNRISSENWPEFKPKTPFGQMPVLEIDGKQYAQSTAICRYLGRKYGLAGANDEEAFEIDQNVEFLNDIRASAASVHYEKDEAVKAKKKAELEETKYPFFFEKLNEILTKNNGHIALGKLTWGDFVYAGMYDYLKAMLQKPDLEQKYPAFRKPIEAVLAIPKVKAYVDAAPRTEL

>BmGSTt1-EF506487

MVLKLYYDLMSQPSRVLYILLKTMKYDFEPKYVNLRKAEHYSEDFTKVNRMQRVPVIDHNGFILTESIAILKYLSRENVIAESLYSKESKLQARIEEFLEWQHIGLRLHCAMYFRVVHMDPILTGRKSDEKTIQGYKRRMMMALDDFDTKWLGRGTAFIVGETPTVADLVAACELEQPRMAGFEPKDHFPNIAAWWPKVRDHFAPHYEDAHVILNKIINKMDRAANSKL

>BmGSTu1-EF423869

MVLKLYAVSDGPPSLSVRQALVALEVPFELINVDFGAGEHMTSDYALMNPQKEIPVLDDEGFYLSESNAILQYICDKYRPGSPLYPQDPKSRAIVNHRLCFNLSSYYANISAYTMAPIFFDYERTPLGLKKVHISLDVLETYLTRTNTSYAAANHLTIADFPLINSTMTLEAIDFDFSKYTKIHKWYNDFKVKYPDLWKISESAMKEIQHFAANPPDLTHLNHPIHPIRKIKN

>BmGSTz1-DQ355375

MGKQPVLYSYWRSSCSWRVRIALNLKEIPYDIKAVSLIKGGGEQHCNEYREVNPMEQVPSLCIDGHTLIESLNIMHYLEETRPQRPLMPQDCFKRAKVREICEMIASGIQPLQNLIVLIYVGEEKKKEWSQHWITRGFRAIEKLLSTTAGKYCVGDEITLADCCLVPQVFNARRFHVDLRPFPIILRIDRELENHPAFRAAHPSSQPDCPPEVAK

>BmGSTz2-EF565386

MVENRVILHAYWLSSCSWRVRAMLHAKSIPFEERPVDIVKTGKQLTEEYRAINPAQKVPALEIDGVTLVESTAIIQYIEDTRPEPKLMPDTALQRARMREICETIVSGIQPLQNFGLKKHLGTEEKFLSFTKYWTERGLQTLNDLLAKTSGAYCIGDQITLADICLVPQIYNGVSRHKLDLKTYPIVSKVYENLLKEELYQATHPKATKEKLKINL

>DmGSTd-CG17639

MSPPVLYYLPPSPPCRSILLLAKMLDIDFELKIVNILEGEQLKPDFVAMNPQHCVPTMNDEGLVLWESRAILSYLVAAYGKSDQLYPTDIRVRALVDQRLQFDLGTLYMRLTDYYFPTMFIGAPLDEGKRAKLAEAVGWLNTILEGRQFSAADHFTIADLTLLVTVSQLEAFEFELRPYKHIRQWLDRCKDHMAPFDYEELNANKANMLADMFKAKMNQSAG

>DmGSTd1-NP_524326

MVDFYYLPGSSPCRSVIMTAKAVGVELNKKLLNLQAGEHLKPEFLKINPQHTIPTLVDNGFALWESRAIQVYLVEKYGKTDSLYPKCPKKRAVINQRLYFDMGTLYQSFANYYYPQVFAKAPADPEAFKKIEAAFEFLNTFLEGQDYAAGDSLTVADIALVATVSTFEVAKFEISKYANVNRWYENAKKVTPGWEENWAGCLEFKKYFE

>DmGSTd10-NP_652713

MDLYYRPGSAPCRSVLMTAKALGVEFDKKTIINTRAREQFTPEYLKINPQHTIPTLHDHGFALWESRAIMVYLVEKYGKDDKLFPKDVQKQALINQRLYFDMGTLYKSFSEYYYPQIFLKKPANEENYKKIEVAFEFLNTFLEGQTYSAGGDYSLADIAFLATVSTFDVAGFDFKRYANVARWYENAKKLTPGWEENWAGCQEFRKYFDN

>DmGSTd2-NP_524912

MDFYYMPGGGGCRTVIMVAKALGLELNKKLLNTMEGEQLKPEFVKLNPQHTIPTLVDNGFSIWESRAIAVYLVEKYGKDDYLLPNDPKKRAVINQRLYFDMGTLYESFAKYYYPLFRTGKPGSDEDLKRIETAFGFLDTFLEGQEYVAGDQLTVADIAILSTVSTFEVSEFDFSKYSNVSRWYDNAKKVTPGWDENWEGLMAMKALFDARKLAAK

>DmGSTd3-NP_788656

MVGKALGLEFNKKIINTLKGEQMNPDFIKINPQHSIPTLVDNGFTIWESRAILVYLVEKYGKDDALYPKDIQKQAVINQRLYFDMALMYPTLANYYYKAFTTGQFGSEEDYKKVQETFDFLNTFLEGQDYVAGDQYTVADIAILANVSNFDVVGFDISKYPNVARWYDHVKKITPGWEENWAGALDVKKRIEEKQNAAK

>DmGSTd4-NP_524913

MDFYYSPRSSGSRTIIMVAKALGLELNKKQLRITEGEHLKPEFLKLNPQHTIPTLVDNGFAIWESRAIAVYLVEKYGKDDSLFPNDPQKRALINQRLYFDMGTLHDSFMKYYYPFIRTGQLGNAENYKKVEAAFEFLDIFLEGQDYVAGSQLTVADIAILSSVSTFEVVEFDISKYPNVARWYANAKKITPGWDENWKGLLQMKTMYEAQKASLK

>DmGSTd5-NP_524914

MDFYYSPRGSGCRTVIMVAKALGVKLNMKLLNTLEKDQLKPEFVKLNPQHTIPTLVDNGFSIWESRAIAVYLVEKYGKDDTLFPKDPKKQALVNQRLYFDMGTLYDSFAKYYYPLFHTGKPGSDEDFKKIESSFEYLNIFLEGQNYVAGDHLTVADIAILSTVSTFEIFDFDLNKYPNVARWYANAKKVTPGWEENWKGAVELKGVFDARQAAAKQ

>DmGSTd6-NP_524915

MDLYNMSGSPSTRAVMMTAKAVGVEFNSIQVNTFVGEQLEPWFVKINPQHTIPTLVDNLFVIWETRAIVVYLVEQYGKDDSLYPKDPQKQALINQRLYFDMGTLYDGIAKYFFPLLRTGKPGTQENLEKLNAAFDLLNNFLDGQDYVAGNQLSVADIVILATVSTTEMVDFDLKKFPNVDRWYKNAQKVTPGWDENLARIQSAKKFLAENLIEKL

>DmGSTd7-NP_525114

MPNLDLYNFPMAPASRAIQMVAKALGLELNSKLINTMEGDQLKPEFVRINPQHTIPTLVDNGFVIWESRAIAVYLVEKYGKPDSPLYPNDPQKRALINQRLYFDMGTLYDALTKYFFLIFRTGKFGDQEALDKVNSAFGFLNTFLEGQDFVAGSQLTVADIVILATVSTVEWFSFDLSKFPNVERWLKNAPKVTPGWEQNLESLQQGKKFLQDLQAAKEKEVKA

>DmGSTd8-NP_524916

MDFYYHPCSAPCRSVIMTAKALGVDLNMKLLKVMDGEQLKPEFVKLNPQHCIPTLVDDGFSIWESRAILIYLVEKYGADDSLYPSDPQKKAVVNQRLYFDMGTLFQSFVEAIYPQIRNNHPADPEAMQKVDSAFGHLDTFLEDQEYVAGDCLTIADIALLASVSTFEVVDFDIAQYPNVARWYENAKEVTPGWEENWDGVQLIKKLVQERNE

>DmGSTd9-NP_650181

MLDFYYMLYSAPCRSILMTARALGLELNKKQVDLDAGEHLKPEFVKINPQHTIPTLVDDGFAIWESRAILIYLAEKYDKDGSLYPKDPQQRAVINQRLFFDLSTLYQSYVYYYYPQLFEDVKKPADPDNLKKIDDAFAMFNTLLKGQQYAALNKLTLADFALLATVSTFEISEYDFGKYPEVVRWYDNAKKVIPGWEENWEGCEYYKKLYLGAILNKQ

>DmGSTe-CG11784

MSKPTLYYALFSPPARACILVAKLIGLDLELKPVDFAKKEHLSEEFVKLNPQHQIPVFVDSDGEVYVDSHAIVCFLVAKYAGNDQLYPRDLKRRAHIDHRMHYENGVLFQVVKDIVARNIYGGEGEYNPRSLTLCHNAYSDLEHFLQQGSFVVGNELSVADVSIHTTLVTLDLLIPVEREKYPQTKQWMERMDKLLPDNEEINLKGARALQTRILSCMAENKAKSQ

>DmGSTe-CG16936

MSKPALYYATLSPPSRAVLLTAKAIGLDLELRPINLLKGEHLTPEFLKLNPQHTIPTLIDGEATIIDSHAICAYLVEKYGQKEQQLYPKELVQRANVDARLHLDSGHLFARLRFLYEPILYYGSTDCSIDKIAYIQKCWEILEGFLKDQPYLCGSDLTIADFCAVATVTSVNDTAPIDEFKFPKMHAWLKRLAELPYYQEVNGDGADELKSIFKAKLAENRGK

>DmGSTe-CG5224

MSAKPILYYAPRSPPCRAVLLTAAALGLELDLRLVNVKAGEHKSAEFLKLNAQHTIPVLDDNGTIVSDSHIICSYLADKYAPEGDDSLYPKDPEKRRLVDARLYYDCGHLFPRIRFIVEPVIYFGAGEVPSDRVAYLQKAYDGLEHCLAEGDYLVGDKLTIADLSCIASVSTAEAFAPIEPDQFPRLVQWVKRIQALPYYQKNNQEGLDMLVGLVKGLLAERQQK

>DmGSTe1-NP_611323

MSSSGIVLYGTDLSPCVRTVKLTLKVLNLDYEYKEVNLQAGEHLSEEYVKKNPQHTVPMLDDNGTFIWDSHAIAAYLVDKYAKSDELYPKDLAKRAIVNQRLFFDASVIYASIANVSRPFWINGVTEVPQEKLDAVHQGLKLLETFLGNSPYLAGDSLTLADLSTGPTVSAVPAAVDIDPATYPKVTAWLDRLNKLPYYKEINEAPAQSYVAFLRSKWTKLGDK

>DmGSTe10-NP_611322

MANLILYGTESSPPVRAVLLTLRALQLDHEFHTLDMQAGDHLKPDMLRKNPQHTVPMLEDGESCIWDSHAIIGYLVNKYAQSDELYPKDPLKRAVVDQRLHFETGVLFHGIFKQLQRALFKENATEVPKDRLAELKDAYALLEQFLAENPYVAGPQLTIADFSIVATVSTLHLSYCPVDATKYPKLSAWLARISALPFYEEDNLRGARLLADKIRSKLPKQFDKLWQKAFEDIKSGAGKQ

>DmGSTe2-NP_611324

MSDKLVLYGMDISPPVRACKLTLRALNLDYEYKEMDLLAGDHFKDAFLKKNPQHTVPLLEDNGALIWDSHAIVCYLVDKYANSDELYPRDLVLRAQVDQRLFFDASILFMSLRNVSIPYFLRQVSLVPKEKVDNIKDAYGHLENFLGDNPYLTGSQLTIADLCCGATASSLAAVLDLDELKYPKVAAWFERLSKLPHYEEDNLRGLKKYINLLKPVLNLEQ

>DmGSTe3-NP_611325

MGKLTLYGIDGSPPVRSVLLTLRALNLDFDYKIVNLMEKEHLKPEFLKINPLHTVPALDDNGFYLADSHAINSYLVSKYGRNDSLYPKDLKKRAIVDQRLHYDSSVVTSTGRAITFPLFWENKTEIPQARIDALEGVYKSLNLFLENGNYLAGDNLTIADFHVIAGLTGFFVFLPVDATKYPELAAWIKRIKELPYYEEANGSRAAQIIEFIKSKKFTIV

>DmGSTe4-NP_611326

MGKISLYGLDASPPTRACLLTLKALDLPFEFVFVNLFEKENFSEDFSKKNPQHTVPLLQDDDACIWDSHAIMAYLVEKYAPSDELYPKDLLQRAKVDQLMHFESGVIFESALRRLTRPVLFFGEPTLPRNQVDHILQVYDFVETFLDDHDFVAGDQLTIADFSIVSTITSIGVFLELDPAKYPKIAAWLERLKELPYYEEANGKGAAQFVELLRSKNFTIVS

>DmGSTe5-NP_611327

MVKLTLYGVNPSPPVRAVKLTLAALQLPYEFVNVNISGQEQLSEEYLKKNPEHTVPTLEDDGNYIWDSHAIIAYLVSKYADSDALYPRDLLQRAVVDQRLHFETGVVFANGIKAITKPLFFNGLNRIPKERYDAIVEIYDFVETFLAGHDYIAGDQLTIADFSLISSITSLVAFVEIDRLKYPRIIEWVRRLEKLPYYEEANAKGARELETILKSTNFTFAT

>DmGSTe6-NP_611328

MVKLTLYGLDPSPPVRAVKLTLAALNLTYEYVNVDIVARAQLSPEYLEKNPQHTVPTLEDDGHYIWDSHAIIAYLVSKYADSDALYPKDPLKRAVVDQRLHFESGVVFANGIRSISKSVLFQGQTKVPKERYDAIIEIYDFVETFLKGQDYIAGNQLTIADFSLVSSVASLEAFVALDTTKYPRIGAWIKKLEQLPYYEEANGKGVRQLVAIFKKTNFTFEA

>DmGSTe7-NP_611329

MPKLILYGLEASPPVRAVKLTLAALEVPYEFVEVNTRAKENFSEEFLKKNPQHTVPTLEDDGHYIWDSHAIIAYLVSKYGKTDSLYPKDLLQRAVVDQRLHFESGVIFANALRSITKPLFAGKQTMIPKERYDAIIEVYDFLEKFLAGNDYVAGNQLTIADFSIISTVSSLEVFVKVDTTKYPRIAAWFKRLQKLPYYEEANGNGARTFESFIREYNFTFASN

>DmGSTe8-NP_611330

MSKLILYGTEASPPVRAAKLTLAALGIPYEYVKINTLAKETLSPEFLRKNPQHTVPTLEDDGHFIWDSHAISAYLVSKYGQSDTLYPKDLLQRAVVDQRLHFESGVVFVNGLRGITKPLFATGQTTIPKERYDAVIEIYDFVETFLTGHDFIAGDQLTIADFSLITSITALAVFVVIDTVKYANITAWIKRIEELPYYEEACGKGARDLVTLLKKFNFTFST

>DmGSTe9-NP_725784

MGKLVLYGVEASPPVRACKLTLDALGLQYEYRLVNLLAGEHKTKEFSLKNPQHTVPVLEDDGKFIWESHAICAYLVRRYAKSDDLYPKDYFKRALVDQRLHFESGVLFQGCIRNIAIPLFYKNITEVPRSQIDAIYEAYDFLEAFIGNQAYLCGPVITIADYSVVSSVSSLVGLAAIDAKRYPKLNGWLDRMAAQPNYQSLNGNGAQMLIDMFSSKITKIV

>DmGSTo-CG6662

MSNTQHLTIGSPKPVFPDDGILKLYSMRFCPYAHRVHLVLDAKKIPYHAIYINLRDKPEWFSLVSSSTKVPALELVKEQGNPVLIESLIICDYLDEKYPEVPLYPKDLLKKAQEKILIERFGQFINAFYYLLLHDNPEQLVDTDHYAGLVVYEEELKRRCTKFFGGDSPGMLDYMMWPWCERFDSLKYTFEQKFELSPERFPTLIKWRDLMIQDRAVKCFYLDGQTHAKYMNSRRSGQADYNMLYNEAKRVKLG

>DmGSTo-CG6673PA

MALPQKHFKRGSTKPELPEDGVPRFFSMAFCPFSHRVRLMLAAKHIEHHKIYVDLIEKPEWYKDFSPLGKVPALQLTGVKDQPTLVESLIIAEYLDQQYPQTRLFPTDPLQKALDKILIERFAPVVSAIYPVLTCNPNAPKDAIPNFENALDVFEVELGKRGTPYFAGQHIGIVDYMIWPWFERFPSMKINTEQKYELDTKRFEKLLKWRDLMTQDEVVQKTALDVQLHAEFQKSKTLGNPQYDIAFKGTP

>DmGSTo-CG6673PB

MALPQKHFKRGSPKPEIPEDGVLRYYSMRFCPYSQRAGLVLAAKKIPHHTVYIDLSEKPEWYIDYSPLGKVPAIQLPNLPGQPALVESLVIAEYLDEQYPGEGSLFPKDPLQKALDRILIERLSPAVSAIYPVLFTKNPPADAIKNFETALDVFEQEITKRGTPYFGGNKIGIADYMIWPWFERFPALKYTLDEPYELDKTRYQNLLKWRDLVAQDEAVKATALDARIHAKFMKTRHENKPDYDVAFQPL

>DmGSTo-CG6776

MSSGKHLAKGSPKPVLPDDGVLRLYSMRFCPYAQRAHLVLNAKNVPYHSVYINLTEKPEWLVEVSPLLKVPALQLVAEKGEPSLIESLIIAEYLDDKYPENPLLPKDPLKRAQDKILLERFSSITSAFINILVQGTGLEDYWTALDIFEEELTKRGTPYFGGNKPGFVDYMIWPWFERLSVIELKLQKEYNFNESRFPKITKWIALLKADSVVQSFYATPEQHNEFWRTRAGNANYDLLA

>DmGSTo-CG6781

MSNGRHLAKGSPMPDVPEDGILRLYSMRFCPFAQRVHLVLDAKQIPYHSIYINLTDKPEWLLEKNPQGKVPALEIVREPGPPVLTESLLICEYLDEQYPLRPLYPRDPLKKVQDKLLIERFRAVLGAFFKASDGGDLEPFWSGLDIYERELARRGTEFFGGEQTGILDYMIWPWCERLELLKLQRGEDYNYDQSRFPQLTLWLERMKRDPAVMAFYMEAEVQAEFLRTRSLGRPNYNLLVKDA

>DmGSTs1-NP_725653

MADEAQAPPAEGAPPAEGEAPPPAEGAEGAVEGGEAAPPAEPAEPIKHSYTLFYFNVKALAEPLRYLFAYGNQEYEDVRVTRDEWPALKPTMPMGQMPVLEVDGKRVHQSISMARFLAKTVGLCGATPWEDLQIDIVVDTINDFRLKIAVVSYEPEDEIKEKKLVTLNAEVIPFYLEKLEQTVKDNDGHLALGKLTWADVYFAGITDYMNYMVKRDLLEPYPALRGVVDAVNALEPIKAWIEKRPVTEV

>DmGSTt-CG1681

MSQPLKFYFDFLNQSSRALYILLEASKIPFEAIPISMLKGEHLTGEFRDNVNRFRKLPAITDHGYQLSENVAIFRHLAREKLVPEHWYPRRHLGRSRIDEYLAWQQTNMGVATTEYFQQKWLVPYLQKTRPADNAVNLASQLEHTLNEFEQLFLNSRKFMMGDNISYADLSAICEIDQPKSIGYNAFQNRNKLARWYETVREELGPHYKEVLGEFEAKLKGSGSGQQQGVAQAVKQ

>DmGSTt-CG1702

MSAPIRYYYDLMSQPSRALFIIFRLSNMPFEDCVVALRNGEHLTEDFKKEINRFQRVPCIHDNGYKLAESVAILRYLSAKGKIPEHLYPKYFVDQSRVDEFLEWQHMSLRLTCAMYFRTVWLEPLLTGRTPSEAKIETFRMQMERNLDVVEEVWLEGKDFLTGSSLTVADIFAACEIEQTRMADYDVRIKYPKIRAWLKRVRQSCNPYYDVAHEFVYKISGTGPQAKL

>DmGSTt-CG30000

MSKAIKYYYDFLSQPSRALWIAMKLGKTPFEDCPVALRKQEQLTDEYRSINRFQKVPAIVDGKFQLGESVSIVRYLADKGVFSEQLYPKTLEERARVDEFLEWQHFNVRLVCSLFFRQVWLLPAKGLAPAPKPESVKKLIKDVESNLGLLERLWLEKDFLVGDKLTVADIFGSSEINQMKLCQYNVNEKQFPKVAKWMERVRDATNPYYDEAHSFVYKTSQQAVKAKN

>DmGSTt-CG30005

MSKPIRFYYDLLSPIARGLWIGLKFSNSPVEYCPIALRKFEQLTDEYKKINRFQKVPAIVGGDFHLSETIAIIRYLADKGQFDEKLYPKTLENRARVDEFLEWQHLNIRLACSMYFRDAWLFPMNGIAPKPKPEQIQALIEGVENNLGLLERLWLENDFLVGKNLTMADILGSSEINQLRLCQYRVDEKKFPKVVKWLERVRVSANPYHDEGLTFIDRKSKQSTAAKL

>DmGSTz-CG9362

MASATQLTHRGIHLAGLYRSSWSKPLFRHLATKPILYSYWPSSCSWRVRVALAIKKIDYDIKPTSLLKTVSGHAYTDEYREVNPMQKVPSLKIDGHTLCDSVAIIHYLEETRPQPALLPQDPVKRAKIREIVELICSGIQPLQNVSVLDHIGKDQSLQWAQHWISRGFQGLEKVLSHSAGKFCVGDELSMADICLVPQVRNARRYKADLTPYPTIVRLNQELQELDVFKATHPSTQPDCPPEFAKK

>DmGSTz-CG9363

MSLSAIAKPILYSYWRSSCSWRVRIAMNLKEIPYDIKPISLIKSGGEQHCNEYREVNPMEQVPALQIDGHTLIESVAIMHYLEETRPQRPLLPQDVHKRAKVREIVEIICSGIQPLQNLIVLIHVGEEKKKEWAQHWITRGFRAVEKALSTSAGKYCVGDEISMADCCLVPQVFNARRFHVDLRPYPIILRIDRELESNPAFRAAHPSNQPDCPPELPNK

>LlGSTd-DQ315381

MTIDFYYTPGSSPCRNVLLAAKAVGVDLNLKLLDLMKGEHLAPDFVKINPQHCVPTLVDNGFVLLESRAIMTYLASKYGKDDSLYPKDPQKRAVVDQRLYFDMGTLYQRFGELYYPIIFGGAPYDEEKAKKLDDAFKFLDGYLGKSEWAAGGNLTVADLALVASVSTAESCDWDVSKYPNVAKWYAKCKTTIPGYAEANQAGADKFKGMYQAAKSK

>LmGST-AEB91972

MAPPTLFNVTLSPPCRLVRLVAGIIGVDLKVVDVKDISKEMKTPEMLKKNPQHTVPTLEDNGVYLAESRAIAMYLISKYAKDDSLYPKDVNKRVLVDQRLFYDQDLYNKILNVFLPKFFGKQTDPSSIEKVNEGLETLNRMLDGKQWLAGDNVTLADYAVAISLSSLDFVPESGIDPKKQPNINQWLPRVENSHPKYGEHLKEFHEALKKLTQK

>LmGSTd1-AEB91971

MPSVDLYYVPGSAPCRAVQMVAKAVGVDLNLKLVNLMEGEQMKPEYLKMNPQHTVPTIDDNGLYLWESRAIIGYLVEQYAKDDSLYPKEAKKRALVNQRMYFDIGTLYARFADYYYPVMFGGASYDPEKLKKLEEAYEFLNKFLEGSDWVAGNSITIADYTIMASVSTAEIIGFDIKKFPKVAAWFEKAKKEIPSYEETNHAGALEFKKLFDSMTAKK

>LmGSTs1-AEB91973

MAPKYKLTYFAIKALGEPIRFLLSYGKIEFEDARLEFEQWPSMKASMPFGQVPVLEIDGKKTWQSLAICRYLGKLVGLAGANDWEDLQIDMAIETVTDLRLKIANFWYETDEAQKAKKKEPLLKEILPFLLPRLDNLVKENGGYLANSKLSWADLYFIGILDYLNFMVEFDLTKDYSNLAALKKKVLEIPAIKEWVDKRPNTEW

>LmGSTs2-AEB91974

MAPKFKLRYGAIKGLGEPVRFLLSYGKIEFEDERIDLQQWPSVKESMPFGQVPVLEIDGKKTWQSLAICRYLGNQIGLAGANDWEDLQIDMAVETVTDLRLKMASFAYENDPSIKEKKKAPVVNESLPFLLGRLDNLVKQNGGHLANGKLSWADLYFVALLDYLNFLTGFDLTKDYPNLSALKRTVLEIPAIKEWVTKRPKTDM

>LmGSTs3-AEB91975

MAPKYKLTYFPIMGLAEPIRFILSYGKIEFEDNRFESDKWPSIKESMPFGQVPVLEIDGKKICQTIAICRYLAKQVGLAGDNDWENLQIDMAADTVTDIRTKIVSPNYETDEEKKQKKKAEVINETLPFLLPRLDNLVKQNGGYLANGKLSWADLYFVALIDLLKFMIGFDITKDYSNLSALKNKVLEIPAIKEWVAKRPKTER

>LmGSTs4-AEB91976

MAPKYKLSYFPIMGLAEPIRFLLSYGKIEFEDERFESDQWASVKPSTPFGQAPVLEIDGKKTWQSVAICRYLGKLVGLAGANDWENLQIDMAVDTISDIRSKIASYGYEADPALKEKKKAAVVNEALPFLLTRLDKLVKDNGGYLANGKLSWADLYFVGILGYTNYVSGLDLTKDYSNLSALKNKVLEIPAIKEWVAKRPKTER

>LmGSTs5-AEB91977

MAPKYKLTYFPIMGLAEPIRFLLSYGKIEFEDERCDQDKWPSVKESTPLGKVPVLEIDGKTTWQSVAICRYLAKQLGLAGANDWEDLQIDMAVDTISDLRMKVAAYAYETDEALKEKKKQSLLNETLPFMLPRLDKMVKENGGYFANGKLSWADLYFAAIPINFMMGFDITKDYSNLSALKNTVCEIPAIKEWISKRPKTER

>LmGSTs6-AEB91978

MESKCKLTYFDAMGTAEPIRYLLYHGKIKFEDNRVDFQTWQSMKTSTPFGQMPVLEIDGKKMHQSIAICRYLGKKLALAGENDWESAQIDMAVDTVLDLRIKLSEFYWESDETVKQRKKATVLNETLPFVLERLNGLVKENGGYLALGKLTWGDFFFAGISEYMNCVTQFDITKDYPNLAALKKKICELPDVKDWISRRPVSQL

>LmGSTs7-AEB91979

MSPTCKLTYFDAMGIGEPIRFLLCYGKIQFEDIRFDFEKWPSMKPSTPYGKVPLLEIDGKKMHQSAAICRYLGKKLGLAGANDWESAQIDMAVDTITDLRLKVTEYYWEPEETTKQKKKETLLNETLPFYMEKLDALVKENGGYLVAGKLSWGDFFFAGVSDYMDCVVQFDITKDHPNLAALKKKIRELPAIKEWISKRPKTTL

>LmGSTt1-AEB91980

MSLKVYYDLLSQPSRAVVLFLLANDIPYEAREINVLHGEQFSEEFAKLNPMKKVPVIKDGDFTLTESVAILRYLCRERDVPDHWYPADSKKQARVDEYLEWQHTNTRSNCALYFLNKFMLPAIKGTQPNPETVARRERKMVATLNEVEEIWLRNKTYLAGDKISIADLLGACEIEQTRMAGYNPCDGRPKLAAWLERVRCDTLPHYDTVHALVRKVTEKYGGVPPASFSKL

>LsGSTd1-JN628446

MAAVTLYHFPYSAPSRGALLAARAVGVPVEIKEINLFEGKHLEADFIKINPQHTIPTLKDGDFVLWDSHAIASYLVSAYGKDDKLYPKNPQQKAIVDQRLYFDVGILYRRVRAIFFPVVRLGEKTVSDEKKKDVEEALGWMDQFLTGRPWLAGTEFTIADCACAASISTLVEMGYDISSHRKTTEWYEKCKAELPGFDENLEGAKILGDIFKQKVDPGQI

>LsGSTe1-JN628441

MTIDFYYMDISPPVRAVNLCLAALNLEVNKKEINLFNRENLEPAFIQLNPQHTIPTIVDDGFVLWDSHAINAYLVSKYAKDDPLYPKDIQKRAIIDQRLHFEGSVLFTHGVRCFLPLFFGLSKTIPDDQRNQTDQYYEMVDKFLEGKPWIAGDQMTIADFSYVSTLSGLTQIFPGVEKYKNICTYMERCKQNMKDYDSANQQGVDKYVGILKNILQSE

>LsGSTo1-JN628448

MAAIEHLTVGSTDPPLVEGKLRLYSMRFCPYAARVHLVLNAKKIPYDPVYINLINKPEWYTSRIPTGKVPALVTEGTDLYESLIIANYLDEKYPENKLQSDDPLRKAKDSILIESFGKVGSIMYKMYFNDIDTETFDQFLGALDDFEKELSSRGTTFYGGNAVKLVDYMLWPFFERMSMFALPDKPQFKIPEARFPCLTKWMTP

>LsGSTs1-JN628440

MAPSRYKLIYFNARGRAEHIRFIFAYAGVEYTDHRIVKEKWPEIKRSTPFGMLPVLEVDGKAVCQSNAVARFLARQYNLAGKDEWEALQCDSLVDTLGDLKQVLWYYRSEQDPIKKEERRATLLKETIPFYLKKFEKVISENGGFSVGNSITWTDFVFAVSLENFELIFGKDSLEPYPHLRKLKERCSLSQALKLGSTRDHKQNSKKINVLN

>LsGSTs2-JN628443

MPTYKLTYFNFSGLGEPIRWLLSYLDVPFEDNRIERENWPTIKSTTPFGQVPVLEVDGKQASQSTAIARYLGKKAGIAGSNEWEDLMIDSMIDTFNDFRMNLVKWFRESDEATKKKLEETLVNETAPFYFNKFNDHIKNNGGYLANGKLSWGDIYFVALLEFMTTIWSGLIDKYEHIKALKEKVVNLPKIKAWIEKRPANVK

>LsGSTs3-JN628444

MSAYKLTYFPVTALGEPIRWLLSYLDIKFEDYRFEREQWPSIKPTTPFGQVPVLEIDGKVVWQSVAISRYFGKKADLAGKDEWEALMIDVIVDTFTDFRMAVGKWFYESDEAAKKKLEIPLFETTVPFYLEKFDSTIKENGGFLANGKLSWGDIFFVAVSGYVNHMLGFNMSDKYENIKALCEKVSAIPKIKEWIDKRPAGI

>LsGSTt1-JN628445

MSTRQSVVTFYYHLLSQPSRALKIFLDRNQIKYIPKEVQLAQGEHLQPEFEAINPFKKVPCISHNGFILTESVAILRYLCREFDVADHWYPKDSLLQARVDEFLEWQHIELRAPLAMYFRTKFLMPMITGKPPNQETVNKMYKMMIVGCDKVENIWLKDKPYLCGNSISIADILGACEIEQPRMAGYDPTEGRPKLNEWMNRIKTDLDPHYADAHTYLNAVIKKNAGKSSENVSKL

>LsGSTz1-JN628442

MSIIGKPVLYSYWRSSCSWRVRIALNLKEIPYDIKPVSLVKGGGEQHCNEFREINPMEQVPALQIDGHTLIESLNIMHYLEETRPQRALMPQDVHKRAKVREICEVIATGVQPLQNLTVLIYVGEEKKKEWAQHWITRGLRAVEKLLSSCAGKYCVGDELTLADCCLVPQVFNARRFHVDLRPFPIALRIDRELENHPAFRAAHPSSQPDCPPEATK

>NlGSTd2-AF448500

MPIDLYYVPGSAPCRNVLLAAKAVGVDLNLKLTDLKSGQHLTPEFIKLNPQHNVPTLDDNGFVLNESRAIMTYLADQYGKDDSLYPKDPKKRAKVNQRLYFDMGTLYQSFGDAYYPHMFGGAPLDEDKKKKLGDALVFLDGFLEKSAFVAGEDLTLADLAIVASISTIEAVEYDLSPYKNINSWYSKVKAAAPGYKEANEEGAKGFGQMFKAMTGK

>NlGSTs1

MSGYKLTYFPVTALGEPIRWMMSYLDIKFEDYRFEREQWPSIKPTTPFGQVPVLEIDGKSVWQSVAISRYFGKKADLAGKDEWESLMIDVIVDTFTDFRLAVGKWFYESDEATKKKLEKPLLETTVPFYLEKFDSTIKENGGFLANGKLSWGDIYFVATSGYINHMLGFNMSDKYENIKALCEKVAAIPKIKEWIDKRPAGI

>NlGSTd1

MAAVTLYHFPYSAPSRGALLAARAVGVPVEIKEINLFEGKQLEPDFIKINPQHTIPTLKDDDFVLWDSHAIASYLVTAYGKDDKLYPRNPQQKAIVDQRLYFDVGILYRRVREIFFPVVRLGEKTVGEEKKKSMEEALGWMDQFLTGRPWLAGTEFTIADCCCAASISTLVEMGYDISSHRKTSEWYEKCKTELPGFDENLEGAKILGDAFKQKVDPGQI

>NlGSTe1

MTIDFYYMDISPPVRAVNLCLAALNLEVNKKEINLFNRENLKPAFIQLNPQHTIPNIADDGFVLWDSHAINAYLVSKYAKDDSLYPKDIQKRAIIDQRLHFEGSVLFTHGVRCFLPLFFGLSKTIPKDQRSQTDQYYEMVDKFLEGNTWIAGDQLTIADFSYISTLSGLSQIFTGVEKYKNISTYMDRCKENMKDYDSANQQGVDKYVGILKNILQSE

>NlGSTo1

MAAIEHLTVGSTDPPLVEGKLRLYSMRFCPYAARVHLVLNAKKIPYDPVFINLMQKPEWYTSKIPTGKVPALVVDGTDLYESLIIANYLDEKYPQNKLQSEDPLKKAKDAILIESFGKVNSVMYKMYFNDIDSETFNQFLDTLDEFEKELSSRGTPFFGGNAVKMVDYMIWPFFERMSVFPLPDRPQFKIPEARFPCLTKWMSAMVEDEAVKQHYLRPDQHAHHLNMRKAGTPDYVIIA

>NlGSTt1

MSGRQSAVTLYYHLLSQPSRALKIFMDVNKIKYIPKEVQLAQGEHLQPEFEAINPFKKVPVISHNNFILTESVAILRYLCREFDVADHWYPKDTLQQAKVDEFLEWQHIELRAPLAMYFRTKFLMPMVTGKPAKQESVDRMYKLMVIGCDKVENIWLKDKPYLCGNSISLADILGACEIEQPRMAGYDPAEGRPKIKEWMDRIKKDLDPHYADAHAYLNAVAKKNAARSSQVVSKL

>NlGSTs2

MPTYKLTYFNFAGLGEPIRWMLSYLDVPFEDNRIEREQWPTIKSTTPYGQVPVLEVDGKQVCQSTAIARYLGKKAGLAGSNEWEDLMIDTMIDTFNDFRSSISKWFKESDEATKKKLEETLLNEKVPFYFNKFNDHIKNNGGYLANGKLSWGDIYFISILEFMTTIWSDIIDKYEHIKALNDKVVNLPKIKAWIEKRPVPKK

>NvGSTd1-NP_001165913

MPVDLYQVPGSAPCRAVRLVAAALGVELNLKHTDLMAKEHLKPEFIKMNPQHSVPTIDDNGFYLWESRAICQYLADKYGKNDSLYPKDPKQRAVVNQRLYFDLGLYQSFADYFYPQIFGGAPADSDKLQKIHDNLKFLETFLDGQKYLAGNNLTIADLLTATTYSNFPFTDVDVSKYKNTAKWFDRVKAEAPKYEQTNGEGLKAFKALIEQLKKK

>NvGSTd5-NP_001165915

MTIDLYYFPPSPPCSSVRMLAKHLGVHLNLKSLNPLKGETMKASFRKINPQHTIPTIDDNGFILWESRPIMAYLVMKYAKNDSLYPKEPESRALVEQRLYFDIGTLYQNILGYHAPVIMGKMDSPPEQLLAPMERAFEVLDGYLQNSQFVAGDELTIADFGIVVSVSLAKGCGFDIGRYDNVAAWHERCKKAMDKYGFEEINEAGSRTLGGFYKANLKA

>NvGSTs2-NP_001165918

MPSYKVTYFNIKGLAEPIRFLLNHGGVDFVDERMNEEDWKRIKPTTPFGQVPILEVDGKKINQSTAICRYLAKQFGLTGKDDWENLEIDAAVDTINDMRVKITSPHYEKVAEAKAQKIEQAKQVVPFILERLEEQVKRNGGYFVGGKLTWADLYFVSLLDMFNFRSGEVDILKNKPHLKALEQKVLQLPNIKAWVEKRPKSDF

>NvGSTs3-NP_001165920

MPTYKLTYFPVKALAEPIRFLFSYAGIEFIDDRFERENWPNIKPTMPFGQVPVLEVDGKKIHQSTAICRWLAKQHGLAGKNDWEALEIDAAVDTIHDVRAKLGAYHYENQSDAKAQKLEVVKEVVPFYIQRLDEQVKKNGGYLVGGALSWADIVFVALLDYLNFMAKYDIIEKAENLKALKEKVLAEPGIKAWVAKRPQSDC

>NvGSTs5-NP_001165922

MPTYKLSYLNVTGLGEPLRFLLSYGGADFEDNRINFEDWPKQKPKMPMEQVPILEFDGKIYHQTRAIGRFLAKKYKLYGNDELQDLEIDLNVDDVEDWRTNFSRFFREADETQKAKLKAFALEKTPFYLGKFEERVKKNGGYFVAGKLSWADVHFAAIAELINNFSEKNNLESYPALTKLYETVRSEPKIKAYLEKRPKTVL

>NvGSTs6-NP_001165923

MPSYKLTYFDITALGEQLRYLLSYGGADFEDCRISLEEWPQLKSKMPMEQLPTLEFDGKVYHQGRAIGRYLAKKYNLYGDDELQALEIDYNVDDIEDWRALFAKIFHETDQSLMAKHKAAVFERTPFFLGKFEERAKKNGGYFVGGKLSWADLHFTGIAELINNFTQRNNLESYPALTKLYGTVRSEPKIKAYLEKRPKTAL

>NvGSTs8-NP_001165924

MPSYKVTYFNIKGLGEPIRFILSQAGVDFVDDRVESADWPKIKPTTPFGQMPVLEVDGKKINQTNAICRYLAKQYGLAGANDWENLEIDATVDTIHDLRAKIGAYYYETNAEAKAEKEKAAKELVPFYVERLDEQVKKNGGYFVGGKLTWADLLFVAVLGYMNGIAKEDIIAKVENLKALREKVLNLPNIKAWVAKRPETEC

>NvGSTt1-NP_001165926

MVLKLYFDFLSQPSRALYILLKTCDIPFEPHILKIALGEHQTEEYEKINPFARLPAIEHDGFKLIESVGIARYLCREFKVPDHWYSASSIQQAKIDEYLEWQHLNTRLYCSRYFTSIVVYAMIRQRAPPPEKEKHFRKDVINCLDTIENVWLKDNRAFIVGDKVSIADIFAACEIEQLRMTEIDPRIGRPKMTAWLDRVATETAPHYALAHRGIDDVATKLKGRQPHTCNFGDIFS

>NvGSTt2-NP_001165925

MSVKFYMDLMSQPSRALYIFMKTTNIPFEKKVTSLKNGENYKDGFEKISPFNKLPVIQHNGFNLTESVAIVRYLAREFNVEDHWYPKDSKAQAKVDEYLEWQHLNTRLHCASYFAVKFLWPIIKGQHIEPKTVVEHEARMIECLDQIENIWLKDNKPFLVGDRITVADLFGACEIEQPRVGGFNPREGRPVLTAWLDRVAKETAPYYEEAHSPMNKVTERNAKQSKL

>NvGSTt3-NP_001165927

MSLKLHYDLLSQPARALYIFLKSCDIPFESNVINLAKREHLQPGYEKINPLRKIPALEHNGFKLTESVAILRYLCREFKVDDHWYPKDSRAQARVDEYLTWQHLNARRPLTTYFRLKYLLPLITGKPTKADKLLELEGEMIESLDLFENIWLKDKPFLAGDKISIADLLGASEVEQPRYAGYDPRDDRPKLTAWLDRVSRETSPFYQEAHANLNEVANKYG

>NvGSTz1-NP_001165931

MSVIGKPILYSYWRSSCSWRVRIALNLKEIPYDIKPISLVKNGGEQHSNEFREINPMEQVPALHIDNHTLIESLNILLYLEETRPHRPLMPVDPVKRARVREICEVIASGIQPLQNLIVLIYVGEERKKEWAQHWITRGLKAVEKLLSASAGKYCVGDEITLADCCLVPQIFNARRFHVDLRPFPTILRVDRHLENHPAFTAAHPNNQPDCPPEATK

>TcGSTs-AY750863

MSSYKVTYFNFTALAEPIRFLLSYLNIDFEDIRFEREQWPALKPNMPFGKVPTLEIDGKVLNQSTAITRYLSKKAGLAGSDDWESLLIDIAVDNIHDLRLAIAGYAYDSNEASKEAKYAPLINETIPFYMDKFEKFVEENNGYFVNGKLSWADLFFVAVLDYLNFMAKVDLLEGRPNLKALKEKVLAVPQIKAWVAKRPTNNP

>SfGSTt1

MSARQSVVTFYYHLLSQPSRALKIFLDHNQIKYIPKEVQLAQGEHLQPEFEAINPFKKVPCISHDGFVLTESVAILRYLCREFIVADHWYPKDSLLQARVDEFIEWQHIELRAPLAMYFRTKFLMPKITGKPPNQATVDKMYKLMVVGCDKVENIWLKDKPYLCGNNISIADILGACEIEQPRMSGYDPTEGRPKLKEWMNRVKTDLDPHYADAHTYLNAIAKKSSGESVGLSKL

>SfGSTo1

MPAIEHLTVGSTDPPPVDGKLRLYSMRFCPYAARVHLVLNAKKIPYDPVYINLITKPEWYTSRIPTGKVPAVVVEGTDLYESLVIANYLDEKYPENKLQVDDPLRKAKDAILIESFGKVGSIMYKMYFNDIDTETFDQFLAALDDFEKELATRGTTFYGGNTVKMVDYMLWPFFERMGSYAVPDKPQFKIPEARFPRLTKWINAMIADEAVKQHYLRPDQHAHHLNMRKAGTPDYVIIE

>SfGSTs1

MSTYKLTYFPVTALGEPIRWLMSYLDIKFEDYRFEREQWPSIKPTTPFGQVPVLEIDGKAVWQSVAISRYFGKKADLAGKDEWESLMIDVIVDTFSDFRLAVGKWFYESDEATKKNLEKPLFETTIPFYLEKFDSKIKENGGFLANGKLSWGDIYFVAVSGYVNHMLGFNMSEKYDNIKALCEKVSAIPKIKEWIAKCPAGI

>SfGSTd1

MAAVTFYHCPISAPSRGALLAARAVGVPVEIKEINLFEGKHLEADFIKINPQHTIPTLKDDDFVLWDSHAIASYLVTAYGKDDKLYPKNPQQKAIVDQRLYFDVGVLYRRVREIFFPIVRLGEKTVSDEKKKAVEEALTWMDQFLTGRPWLAGTEFTIADCSCAASVSTLVELGYDISSHRKTSEWYEKCKTELPGFDENLEGAKILGDIFKQKVDPGQM

>SfGSTe1

MTIDFYYMDISPPVRAVNLCLAALNLEVNKKEINLFNRENLEPAFIQLNPQHTIPTIVDDGFVLWDSHAINAYLVSKYAKDDSLYPKDIQKRAIIDQRLHFEGSVLFTHGVRCFLPLFFGLSKRIPDDQRNQTDQYYEMVDKFLEGKSWIAGDQMTIADFSYISTLSGLTQIFSGVEKYKNICAYMERCKDNMKDYDTANQQGVDKYVGILKNILQSE

>SfGSTd2

MPIDLYYVPGSAPCRNVLLAAKAVGVDLNLKLTDLKSGQHLTPEFIKLNPQHNVPTLDDNGFVLNESRAIMTYLADQYGKDDSLYPKDPKKRAKVNQRLYFDMGTLYQSFGDAYYPHMFGGAPLEEEKKKKLGDALVFLDGFLEKSPFVAGENLTLADLAIVASISTIEAVEYDLSPYKNINSWYAKVKAAAPGYKEANEEGSKAFGQMFKAMTGK

>SfGSTz1

MSIIGKPVLYSYWRSSCSWRVRIALNLKEIPYDIKPVSLVKGGGEQHCNEFREINPMEQVPALQIDGHTLIESLNIMHYLEETRPQRALMPQDVHKRAKVREICEVIATGVQPLQNLTVLIYVGEEKKKEWAQHWITRGLRAVEKLLSSCAGKYCVGDELTLADCCLVPQVFNARRFHVDLRPFPITLRIDRELENHPAFRAAHPSSQPDCPPEATK

>LsGSTm-JN628447

MMSSNLYTTDNPVFSAYLFYCAILVLKVLLMAPLTGRYRFTKRIFANPEDKLPRSIVKYDDPDIERVRRAHLNDLENIPVFMVAALLYIATKPSYWLALNLFRTFTIARIIHTLVYAVVVIPQPARALAWVVGYAATVYIAVQVILFSLIKY

>NlGSTm2

MSSSLYTTDNPVFSAYLFYCAILVLKVLFMAPLTARYRFAKRIFANPEDTTLTPKSKVKYDDLDIERVRRAHLNDLENIPVFMVAALLYIATNPSYFLAVNLFRIFTIARIIHTFVYAIV

VIPQPARALAWGVGYAATIYLALQVVLFSL

>NlGSTm1

MSSNLYTSDNPVFSAYLFYCAILVLKVLLMAPLTGRHRFAKRIFANPEDKLPKSVVKYDDPDIERVRRAHLNDLENIPVFMVAALLYIATNPSYWLALTVFRVFTVARIVHTIVYAVVVVPQPARAIAWGVGYAATIYLAVQVILFSL

>SfGSTm1

MSSNLYTTDNPVFSAYLFYCAILVLKVLLMAPLTGRHRFAKRIFANPEDRLPRSIVKYDDPDIERVRRAHLNDLENIPVFMVAALLYIATKPSYWLALNLFRAFTVARIIHTFVYAVVVIPQPARALAWFVGYAATIYIAVQVILFSL

>SfGSTm2

MSSSLYTTDNPVFSAYLFYCAILVLKVLFMAPLTARQRFSKRIFISPEDTTLTPKAKVKHDDPDIERVRRAHLNDLENIPVFMVAALLYIATNPAYFLAVNLFRIFTIARIIHTFVYAVVVIPQPARALAWGAGYAATIYVAVQVILFSL
