# Supplementary figures and images for: Genomic Insights into the Glutathione S-Transferase Gene Family of Two Rice Planthoppers, Nilaparvata lugens (Stål) and Sogatella furcifera (Horváth) (Hemiptera: Delphacidae)
Source: PLoS One. 2013 Feb 14;8(2):e56604. doi: 10.1371/journal.pone.0056604 (PMC3572974; doi:10.1371/journal.pone.0056604)

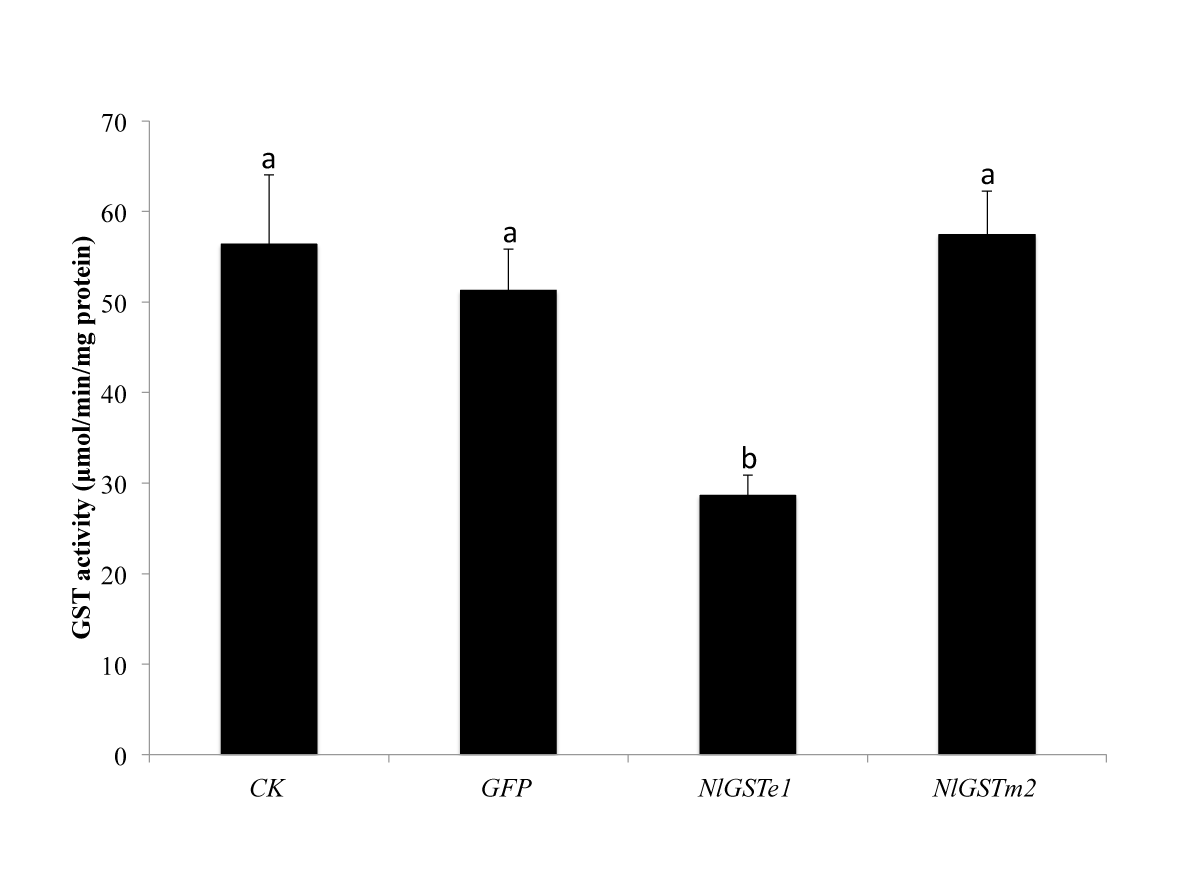

Supplement: Figure S1 — Effect of dsRNA on GST enzyme activity. Results of GST enzyme activity are expressed as µmol (GSH) min−1 mg−1 protein. CK, nymph without any injection; GFP, nymph injected with dsRNA of Green fluorescent protein (GFP) gene; NlGSTe1, nymph injected with dsRNA of NlGSTe1; NlGSTm2, nymph injected with dsRNA of NlGSTm2. Values with different letters are significantly different as determined using a one-way ANOVA (Duncan's multiple range test, P<0.05). (TIF) [file pone.0056604.s005.tif]

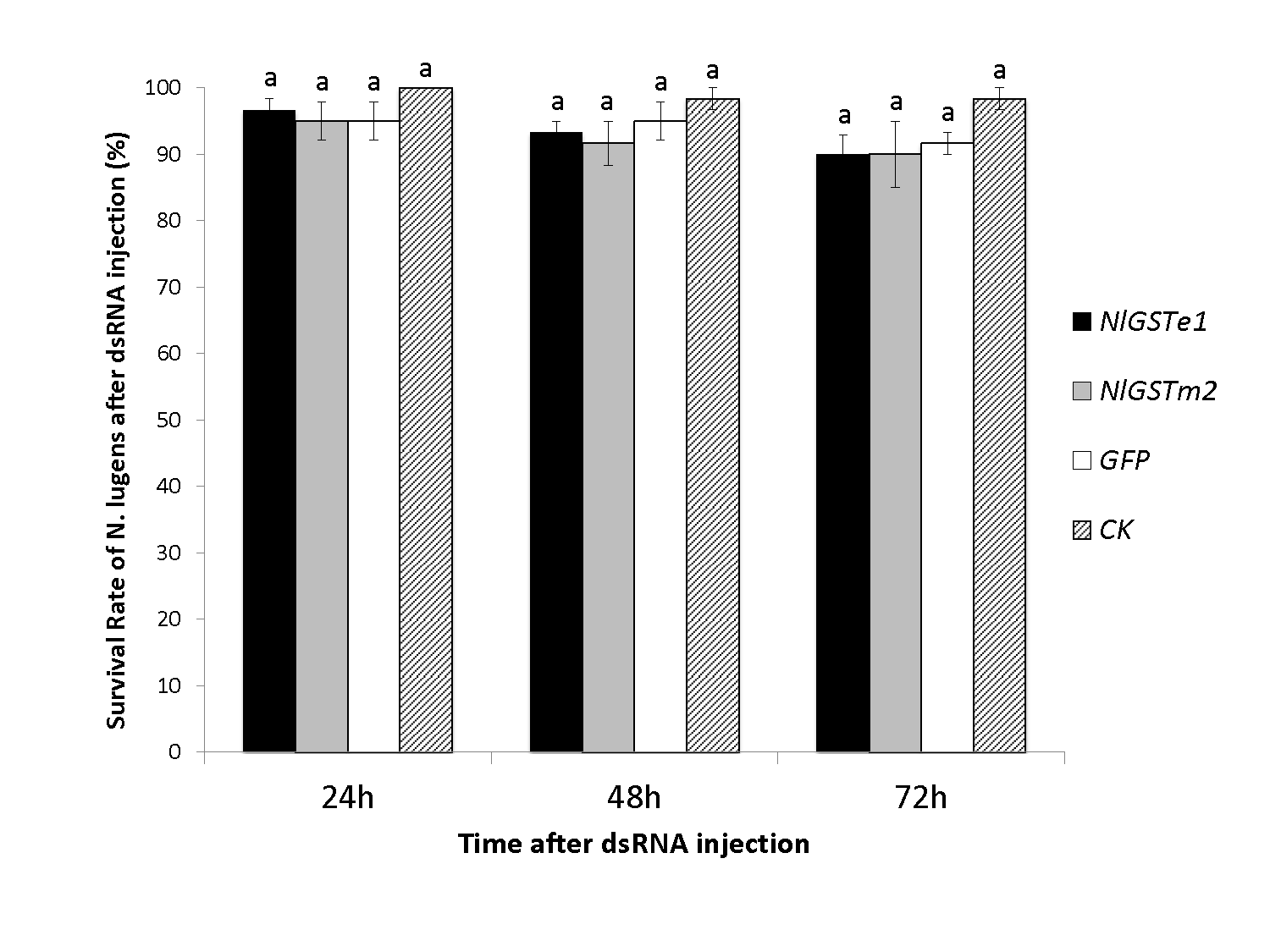

Supplement: Figure S2 — Survival rate of Nilaparvata lugens after dsRNA injection. CK, nymph without any injection; GFP, nymph injected with dsRNA of Green fluorescent protein (GFP) gene; NlGSTe1, nymph injected with dsRNA of NlGSTe1; NlGSTm2, nymph injected with dsRNA of NlGSTm2. Values with different letters are significantly different as determined using a one-way ANOVA (Duncan's multiple range test, P<0.05). (TIF) [file pone.0056604.s006.tif]
